# Ergodicity transformations predict human decision-making under risk

**DOI:** 10.1371/journal.pcbi.1014409

**Published:** 2026-07-20

**Authors:** Benjamin Skjold, Simon Richard Steinkamp, Colm Connaughton, Oliver James Hulme, Ole Peters

**Affiliations:** 1 London Mathematical Laboratory, London, United Kingdom; 2 Danish Research Centre for Magnetic Resonance, Department of Radiology and Nuclear Medicine, Copenhagen University Hospital - Amager and Hvidovre, Copenhagen, Denmark; 3 Warwick Mathematics Institute and Centre for Complexity Science, University of Warwick, Coventry, United Kingdom; 4 Department of Psychology, University of Copenhagen, Copenhagen, Denmark; 5 Santa Fe Institute, Santa Fe, United States of America; University of Birmingham, UNITED KINGDOM OF GREAT BRITAIN AND NORTHERN IRELAND

## Abstract

Decision theories commonly model human behavior as maximizing the expected value of a utility function. This function may vary from one person to another but is assumed to be stable over time. Recent theoretical developments demonstrate that these assumptions are generally incompatible with growing wealth at the fastest rate. Growth optimality requires utility functions to mirror ergodicity transformations and adapt to the dynamic environment. We exposed human participants to different wealth dynamics in a consequential risky decision-making experiment. Via Bayesian modelling, we estimated utility functions separately for each dynamic. Pre-registered analyses revealed strong evidence supporting the quantitative predictions of the ergodicity model. Our study provides evidence that human risk-taking can adapt quickly to the dynamical context, in ways that align closely to the theoretical optimum for maximizing wealth over time.

## Author summary

Why do people sometimes take risks and other times play it safe? Classic decision theories often assume each person has a stable “utility function”—a fixed way of valuing gains and losses—and that people choose options that maximize expected utility. Recent theory suggests this picture can fail when the real goal is to grow resources over time. In particular, the strategy that maximizes long-run growth can depend on how wealth changes: sometimes gains and losses add up (additive dynamics), while in other settings outcomes compound (multiplicative dynamics). Growth-optimization theory predicts that the utility function that best describes behavior should therefore shift with the underlying wealth dynamics. We tested this idea in a fully consequential risky decision-making experiment in which participants experienced different wealth dynamics and their wealth was updated on every trial. Using Bayesian models, we estimated participants’ utility functions separately under each dynamic. We found strong evidence that people change their risk-taking under these different conditions. These results suggest that human risk-taking is not governed by a single fixed utility function, but can adapt rapidly to the dynamical context in a way consistent with long-run growth optimization.

## Introduction

A central theme in the decision sciences is how people behave in risky situations. Theoretical work in this area has produced various quantitative models, which are assessed empirically through field observations, questionnaires, and experimental tasks. Experiments typically involve a form of gambling with real monetary incentives and are designed to test the validity of theoretical assumptions. An open question is whether people’s appetite for risk is dominated by adaptations to the environment or by trait-like differences between individuals.

One model comes from expected utility theory [1–4] in which a transformation—the utility function—is applied to wealth, and people’s decisions are modelled as maximising the expected value of the transformed wealth (expected utility). The utility function expresses trait-like risk preferences, which are free to vary from individual to individual but are stable over time within individuals. This predicts that utility functions are stable features of the individual and are not changeable in response to different environments. This is a specific interpretation of expected utility theory, which we adopt in this paper and refer to as the “EUT model.”

A different model based on ergodicity economics focuses specifically on what happens over time when decisions are made. Here, people are modelled under the assumption that they prefer their wealth to grow faster over time [5] and thus maximise the time average growth rate of wealth. In this particular model, this is also implemented by applying a transformation—the ergodicity transformation—to wealth and modelling people as maximising the rate of change of transformed wealth. Depending on the dynamical environment, different transformations are required: If the dynamical environment in which the decisions are made is additive, then this transformation is linear, whereas a multiplicative environment requires the transformation to be logarithmic. Other dynamics would correspond to different ergodicity transformations [6–8]. The ergodicity transformation is thus identical for all individuals but adaptive to the dynamical environment. We refer to this as the “EE model.”

As the two models make different quantitative predictions for how people make decisions under risk, it is possible to experimentally distinguish between them with the right experimental design. While risk preferences have been extensively studied in experimental settings, in almost all cases, participants are asked to select a single lottery to be realised, make a choice in a series of one-shot lotteries, or set a price at which they would be willing to sell a given one-shot lottery. A general finding that is widely agreed upon is that most subjects are moderately risk averse but that there is still substantial heterogeneity across subjects when tested under laboratory conditions [9–11]. Given that almost all experiments assess risk preferences based on one-shot lotteries, the dynamic by which a participant’s wealth is updated cannot be unambiguously inferred, making it impossible to predict which ergodicity transformation participants should use. An exception is an experiment from Haghani and Dewey [12], who tasked participants with playing a multi-round game with multiplicative dynamics, in which they could invest a variable proportion of their endowed wealth in a series of rounds. This is a way of testing whether they behave according to the so-called Kelly criterion [13], a strategy that maximises the time average growth rate of wealth under games with multiplicative dynamics. The mean proportion recorded (∼ 15%) was relatively close to the optimal proportion for growing wealth at the fastest rate (20%), though with a large variance both within and across participants. However, as this experiment did not manipulate gamble dynamics, it does not offer causal evidence for whether this is an adaptation to the dynamical environment or not.

To our knowledge, there are currently two risk-taking studies that experimentally control dynamics [14,15]. In the experiment by Meder and colleagues [14], participants learnt, in separate additive and multiplicative sessions, what dynamical effect different image stimuli had on their wealth. They subsequently chose gambles based on these images, allowing the authors to estimate how the dynamics influenced their risk-taking behaviour. They found that, as predicted by the EE model, the estimated risk aversion parameter increased under multiplicative dynamics, distributing close to the values that maximise the time average growth rate of wealth. The study was intensive in that participants made over 600 choices but small in participant sample size (*n* = 18) and was criticised for having potential confounds between the dynamical conditions in terms of ambiguity of the outcomes, anticipated wealth levels, and bounds on wealth levels, which may have affected the conditions differently [16,17]. In a separate experimental study, Vanhoyweghen and colleagues [15] found that participants increased their risk aversion under multiplicative relative to additive dynamics, but only under the condition of time-pressured decisions, and thus similar to [14]. This result was interpreted to extend the findings of Meder and colleagues [14]; however, see [17] for a critical discussion.

Because the qualitative prediction has been tested previously, the contribution of the present study is not a new effect, but a stronger and more diagnostic test of the same core mechanism under cleaner experimental control. Specifically, we treat this work as a preregistered, higher-powered conceptual replication and principled generalization of [14], designed to reduce ambiguity about what participants experience and to tighten the mapping between the theoretical intervention (wealth dynamics) and observable choice. To this end, the present design addresses methodological concerns raised about earlier implementations, including potential differences between conditions in anticipated wealth, ambiguity about trial-wise wealth trajectories, and the degree to which choices are consequential. Here, wealth is displayed and updated directly during learning and decision-making, and decisions in the main task have immediate trial-by-trial consequences for in-game wealth, with payoffs proportional to final in-game wealth. In addition, learning and decision demands are matched as closely as possible across dynamics, and identifiability of parameters and models was validated via extensive simulation-based recovery (Appendix C, Fig E in S1 Text). Together, these changes sharpen quantitative model adjudication between the EUT and EE accounts relative to prior work. More broadly, given ongoing concerns about reproducibility in behavioural and psychological science, we view preregistered replication and quantitative sharpening of theoretically constrained, counterintuitive predictions as an important contribution—especially when they bear on foundational assumptions such as the stability of utility.

Here, we employed an experimental design similar to that of Meder and colleagues [14]. The logic is the same: participants made a series of decisions between different gambles taking place under different dynamical environments (specifically, additive and multiplicative dynamics) and ask whether a single idiosyncratic function (the EUT model) or two different dynamic-dependent functions (the EE model) better explains their observed behaviour. The experimental design and planned analyses were preregistered prior to data collection [18]. Participants first completed two sessions corresponding to the two different conditions. Each started with a learning task followed by a decision task. In the learning task, participants observed a sequence of images and the changes in wealth caused by these images. In the subsequent decision task, they chose between gambles represented by two pairs of these images. Repeating this procedure in separate sessions with either additive or multiplicative wealth dynamics, we assessed the effect of dynamics on risk-taking behaviour. Our main question, therefore, is: Which model explains the observed behaviour better, the EUT model or the EE model? To do this, we chose a specific family of functions, known as the isoelastic family (see eq. 4) and fitted a single risk aversion parameter, η (the dependent variable), in the additive and multiplicative dynamical environments (the independent variable). This parameter indicates the propensity of an agent to substitute between the mean and the variance of a gamble. Agents with a parameter value of zero give no weight to the variance of the gamble, whereas agents with a parameter value below zero favour higher variance gambles and agents with values above zero favour lower variance gambles. Fitting such functions to the data allowed us to compare models quantitatively, with the EUT model predicting $\eta^{\text{add}}=\eta^{\text{mul}}$ and the EE model predicting $\eta^{\text{add}}=0$ and $\eta^{\text{mul}}=1$. Using an optional stopping procedure, our pre-registered evidence threshold was reached in support of the EE model after 58 participants, implying that the dynamical intervention exerts a strong and systematic effect on risk-taking behaviour.

## Methods

### Ethics statement

Our study protocol and data collection were approved by the Committee on Health Research Ethics for the Capital Region of Denmark (reference no. H-22046090) and complied with the Declaration of Helsinki. Informed written consent was obtained from all participants.

### Participants

**Recruitment.** Participants were recruited according to the following criteria. Inclusion criteria: Young, healthy adults (18–50 years old); fluent in English; expecting a fixed income three months before and after the experiment. Exclusion criteria: low credit score (self-checked status on www.dininfo.dk); history of psychiatric or neurological disorder; history of drug abuse; training in psychology, finance, accounting, mathematics, physics, engineering, computer science; any other expertise in finances or quantitative science; under medication or medical supplements at the time of the experiment. The experiment took place in Copenhagen, and thus, the participants were primarily residents of Copenhagen.

**Compensation and payoffs.** Participants were compensated for their time and received additional performance-related payoffs. The compensation for participation was DKK 500 (∼USD 75); the performance-related payoff could range from DKK 0–5000 (∼USD 0–730), with an average of DKK 500 (∼USD 75). Participants were truthfully informed that their ability to learn the effect of the images in the learning task and then choose which gambles to take in the decision task would influence their in-game wealth and that this would determine their payoff in real money (see Appendix A).

**Optional stopping and exclusions.** As specified in the pre-registration of the experiment [18], we stopped collecting data once a Bayes factor evidence threshold of 10 was surpassed, corresponding to strong evidence according to the standard interpretation of Bayes factors [19], with a minimum of 50 participants to afford exploratory analyses and a maximum of 150 participants due to resource constraints on payoffs and fees. These numbers were motivated by a Bayes factor design analysis [20, 21, BFDA] for a Bayesian paired t-test with a medium effect size (Cohen’s d, ξ=0.5; see Appendix B). Within the constraints on participant numbers, 100% of studies hit the H1 boundary. For a zero effect size, 71.8% of studies correctly hit the H0 boundary (1.6% incorrectly hit the H1 boundary, and 26.6% terminated inconclusively at 150 participants; panel a of Fig A in S1 Text). 90% power, defined as the probability of accepting H1 over H0 given that H1 generated the data (with a medium effect size), is achieved after 69 participants (panel b of Fig A in S1 Text). We recruited 68 participants in total, of which 10 participants were excluded according to our exclusion criteria. This includes two wrongfully included participants who did not meet our inclusion criteria and eight participants who did not meet our minimum performance requirement in the no-brainer task (see below). Data from 58 participants (25 females) aged 18–48 (mean = 26.37, sd. = 6.84) was included in the analyses.

### Experimental protocol

**Test day.** Participants were tested in parallel in groups of up to 15 using identical standardised laptops. After arriving, participants were instructed to read the information sheet (see full information sheet in the experiment folder under participant_information). In brief, participants were truthfully informed that the experiment aimed to study decision-making under risk. Before each task, game instructions were shown on the screen in a slideshow-like format, allowing participants to navigate back and forth (see instruction slideshow in the experiment folder under instructions). This automated procedure was implemented to ensure participants had time to understand the game and to minimise the biasing of participants by instructors. The code for running the experiment can be found on osf.io (see Data availability) and uses PsychoPy3 [22, v2021.2.3]. Each session began with a learning task in which participants repeatedly observed the effect each of the nine different images had on their wealth. We used abstract fractal images from a validated corpus [23, see Appendix B]. The learning task was followed by a decision task where participants made gambling decisions based on what they just learnt. The experiment comprised two sessions, one for additive and one for multiplicative dynamics (described in the Learning task section below). After completing the first session, participants had a mandatory break (>10 mins) before starting the second session.

**Questionnaires.** After completing both sessions, participants completed a set of questionnaires. These included the Risk Propensity Scale (RPS) [24], and the revised 30-Item Domain-Specific Risk-Taking (DOSPERT) Scale [25], using all three scales (risk-taking, risk perception, and expected benefits).

**Fig 1:**
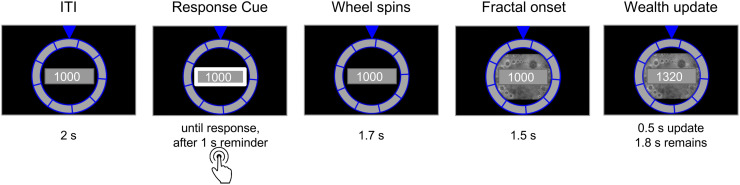
Trial structure for the learning task. The individual steps in each trial, including timings, within the learning task. In each trial, participants respond to a cue, triggering the wheel to spin. Next, the fractal appears, and the wealth is updated. This way, participants learn to associate the image with changes in their wealth.

**Learning task.** The learning task began with 45 learning trials. Wealth was initialized at 1000 points and displayed centrally on the screen at all times. A learning trial consisted of the following steps (Fig 1): To begin each trial, the participant had to press the spacebar. The screen then showed a spinning wheel, indicating the impending random selection of an image. Next, one image, $\omega_{i}$, out of a space Ω of nine different images, was shown inside the wheel for 1.5s. Next, with the image still showing, the participant’s wealth changed. The image remained visible for another 1.8s and then disappeared. After 2s, the participant was prompted to start the next trial. The participants learned to associate each image with a corresponding change in wealth. In the additive session, each image caused a fixed additive change in wealth. For example, one image always caused an increase of 309.5 points in wealth; another always caused a decrease of 49 points, and so on. In the multiplicative session, each image caused a fixed multiplicative change in wealth. For example, one image caused an increase in wealth by a factor of 1.184; another caused a decrease by a factor of 0.649, and so on. Within each run, the 45 trials were generated by sampling independently and uniformly at random (with replacement) from the nine images assigned to that session, so images appeared in interleaved order (not blocked by image) with ~5 presentations per image per run in expectation. Each session comprised three such runs, yielding 135 learning trials per session and ~15 presentations per image on average. The additive and multiplicative conditions were run as separate sessions with independent nine-image sets ($\Omega_{\text{add}},\Omega_{\text{mul}}$) and a mandatory >10-minute break between them, i.e., the two dynamics were not interleaved within a single session.

**Fig 2:**
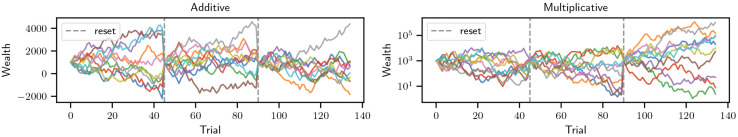
Wealth fluctuations from the learning task. Wealth trajectories from the learning task from 10 randomly selected participants in each session. Note that wealth is on a linear scale for the additive session and a logarithmic scale for the multiplicative session. Wealth is reset twice (after 45 trials) during each learning task.

Independently for each participant, we randomly assigned nine images to the additive session ($\Omega^{\text{add}}$) and nine images to the multiplicative session ($\Omega^{\text{mul}}$). For a given participant, each image always had the same dynamical effect on the participant’s wealth. Sequences of images were randomly generated, causing stochastic fluctuations in wealth (Fig 2). Denoting the trial by t∈{1,…,45}, the image shown as ω(t), the corresponding wealth change by δx[ω(t),x(t−1)], wealth after the trial by *x*(*t*), and initial wealth by *x*(0), we have


$$\begin{array}{c} x(t)=x(t-1)+\delta x[\omega (t),x(t-1)]. \end{array}$$ (1)

To be explicit, in the additive session the wealth change was independent of current wealth:


$$\begin{array}{c} \delta x[\omega (t),x(t-1)]=\delta x(\omega (t)),\;\delta x(\omega )\in \{-407.0,-305.5,-241.5,-144.5,-49.0,108.5,210.5,309.5,440.5\}. \end{array}$$

In the multiplicative session the wealth change scaled with current wealth:


$$\begin{array}{c} \frac{\delta x[\omega (t),x(t-1)]}{x(t-1)}\in \{0.427,0.583,0.649,0.841,1.006,1.184,1.446,1.739,2.164\}. \end{array}$$

The sequence of images was generated by drawing each image with equal probability for each trial (with replacement). These increments and factors were designed via extensive simulation to be experimentally informative for estimating the risk aversion parameter (eq. 4).

**Fig 3:**
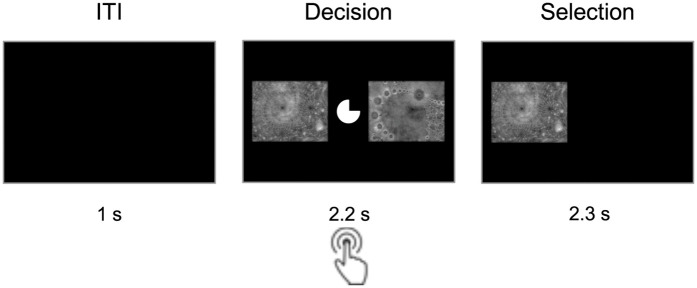
Trial structure for the no-brainer task. The individual steps in each trial, including timings, within the no-brainer task. Participants are tasked to decide between two images which one they think is better for their wealth. The pie chart indicates the amount of time left to choose.

**No-brainer task.** After 45 learning trials, the participants performed 15 no-brainer decisions allowing us to monitor the progress of learning. These no-brainer trials were a minimal effort test of how well the participants had learnt the effect of each image. In each trial, participants were asked to “Choose the fractal you think is better for your wealth.” They had to press either the left or right key to choose. A no-brainer trial consisted of the following steps (Fig 3): The fractals appeared on the screen, and participants had 2.2s to respond by pressing left or right using the keyboard arrows; the remaining time was indicated by a pie chart in the middle of the screen. After a response was issued, the selected image remained on the screen for 2.3s. There was no feedback to the participants from their responses, and their responses did not influence their wealth. If participants did not respond in time, a reminder to press earlier was shown instead. After 1s, the next trial began. Once the no-brainer trials were completed, wealth was reset to its initial value of 1000 points, and a new sequence of 45 learning trials, followed by 15 no-brainer trials, began. This pattern of learning trials, then no-brainer trials, occurred three times. Participants who performed poorly in the no-brainer trials (<80% correct responses) were excluded from further analyses. The data shows that learning proceeded quickly, and most participants (58/66) exceeded 80% correct responses in the last run. Note that no-brainer trials are easily solvable without tracking absolute wealth: within a session the ranking of images is monotonic in wealth (so learning the ordering over images is sufficient), and no-brainer responses do not update wealth. A direct learning-reliability check (no systematic difference in no-brainer accuracy between conditions) confirmed that this was learnt by participants (Appendix D, Learning reliability checks and Fig L in S1 Text).

**Fig 4:**
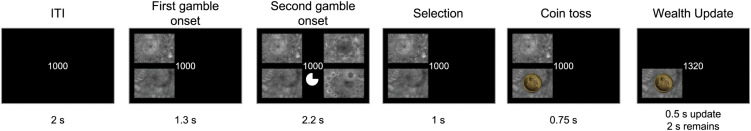
Trial structure for the decision task. The first gamble is always presented on the left, followed by the second gamble on the right. The pie chart that onsets with the second gamble indicates the amount of time left to choose. At the end of this period, the selected gamble persists on the screen, here being the left gamble. Then, a coin toss determines which fractal is applied to wealth; here, it is tails, so it is the lower fractal that is applied to the participant’s wealth. At this point, the wealth is updated, and the next trial is ready to begin.

**Fig 5:**
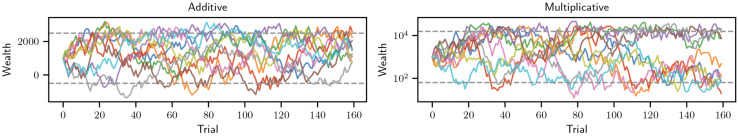
Wealth fluctuations from the decision task. Wealth trajectories from the decision task from 10 randomly selected participants in each session, linear scales for the additive session, and logarithmic scales for the multiplicative session. Horizontal dashed lines indicate bounds beyond which control measures were implemented to keep wealth within a reasonable range (see Appendix **B)**.

**Decision task.** In the decision task, wealth was initialized at 1000 points and again displayed in the centre of the screen at all times. A decision trial consisted of the following steps (Fig 4): A screen with wealth in the centre was displayed for 2s; next, a pair of images was displayed for 1.3s on the left, after which another pair of images was added on the right. Each pair of images represented a gamble, namely a fair coin toss for the wealth change associated with each image. The participant had 2.2s to choose one of the gambles, left or right. A pie chart in the centre of the screen indicated the time left to decide. Once the choice was made, only the two images of the chosen gamble remained on screen. To indicate the outcome of the gamble, a coin appeared on top of one of the images (heads for the top image, tails for the lower, each with probability 1/2). The participant’s wealth was then updated according to this image. If the participant failed to select a gamble within the allowed time (2.2s), then the image that caused the worst wealth change was automatically selected and applied to their current wealth. 160 decision trials were performed, sampling different pairs of gambles from a specific space of possibilities. In Fig 5, we show wealth trajectories for ten randomly selected participants.

We denote the gambles on offer within a given trial by $\gamma_{\text{L}}$ and $\gamma_{\text{R}}$. Participants chose between them via the left and right arrow buttons. Denoting the images constituting $\gamma_{\text{L}}$ by $\omega_{\text{L}}^{\text{top}}$ and $\omega_{\text{L}}^{\text{bottom}}$, the left gamble can be expressed formally as a wealth change δx randomly generated according to:


$$\begin{array}{c} \delta x_{\text{L}}=\left\{ \begin{array}{cc} \delta x(\omega_{\text{L}}^{\text{top}}) & \text{ with probability 1/2}\\ \delta x(\omega_{\text{L}}^{\text{bottom}}) & \text{ with probability 1/2} \end{array}\right. \end{array}$$ (2)

and equivalently for the right gamble, $\gamma_{\text{R}}$.

Offering a choice between two gambles was chosen to eliminate any confounds caused by potential preferences for or against gambling. Offering equal probabilities was chosen to eliminate probability distortion effects and was easily conveyed to the participant by the term “fair coin toss.” The space of possible gambles for each session, Γ, comprised all possible pairs of the nine images in the session-specific Ω, irrespective of order and excluding self-pairing, $\Gamma =\{(\omega_{i},\omega_{j})\in \Omega^{2}|1\leq j<i\leq 9\}$. Hence, there were $\frac{9\times (9-1)}{2}=36$ different possible gambles within each session, |Γ|=36. The choice offered to a participant was always between a pair of gambles, which we denote as ζ(t). Thus, we had gamble pairs (denoted ζ), which consisted of gambles (denoted γ), which in turn consisted of images (denoted ω):


$$\begin{array}{c} \underset{\zeta (t)}{\underbrace{\left[\underset{\gamma_{\text{L}}}{\underbrace{\left(\omega_{L}^{\text{top}},\omega_{L}^{\text{bottom}}\right)}};\underset{\gamma_{\text{R}}}{\underbrace{\left(\omega_{R}^{\text{top}},\omega_{R}^{\text{bottom}}\right)}}\right]}}. \end{array}$$ (3)

The space of possible pairs of gambles *Z* (disregarding order and excluding gambles comprised of identical images) consisted of $\left|Z\right|=\frac{36\times (36-1)}{2}=630$ gamble pairs. We selected a subset of this space to focus on the gamble pairs most informative for distinguishing between the two models. To this end, we eliminated all gamble pairs where one gamble state-wise dominated the other [26, “at least one of the outcomes is better, and none are worse”]. This restricted the gamble pair space to 126 unique gamble pairs in each condition. If wealth became too high or too low, the participant’s choices would no longer be informative for distinguishing between the two models using the nine images. For this reason, we introduced wealth control mechanisms to have as many choices in an informative range with minimal perceived intervention (see Appendix B).

### Design

**Experimental design.** The experiment was a fully crossed, repeated measures, randomised controlled trial in which the type of wealth dynamic (additive or multiplicative) was the primary independent variable, and gamble choices in the decision task were the primary observable data. It was primarily a within-participant design in which two different wealth dynamics were assigned to the same participant in a randomised order.

**Blinding.** The order of the dynamic and images used within each dynamic were randomly assigned to the participants via the coded paradigm, and the experiment was thus double-blinded. Participants were not informed of explicit details concerning wealth dynamics or of any possible differences between sessions. The procedures and setup were identical regardless of the dynamic.

**Validation.** We validated our risk aversion parameter estimations numerically by confirming that we can accurately recover risk aversion parameters from choices made by synthetic agents with known parameters spanning a large range of values (−0.5, 0.0, ..., 1.5), using 160 decision trials per session as implemented in this experiment. These simulations show neither floor nor ceiling effects (see Appendix C). For robustness, the range of simulated parameter values was substantively broader than typically empirically observed [9,14].

### Models

**Framework.** We work with a descriptive framework often used in decision science. Here, future wealth is modelled as a random variable, *x*, and agents optimise the expected value of a function of this random variable; that is, they optimise ⟨f(x)⟩. In the EUT model, this function is called the utility function and models idiosyncratic differences in risk preference [1, summarised in 27]. In the EE model, this function is called the ergodicity transformation, and it is dictated by the dynamics via the assumption that ergodic growth rates are to be optimised over time [5–7]. We restrict ourselves to a specific family of functions, known as the isoelastic family [28], parameterised by η, and given by:


$$\begin{array}{c} f_{\eta }(x)=\left\{ \begin{array}{cc} \frac{x^{1-\eta }-1}{1-\eta } & \text{for}\;\eta \text{≠}1\\ \ln x & \text{for}\;\eta =1. \end{array}\right. \end{array}$$ (4)

We illustrate the relationship between the risk aversion parameter and the effect this has on risk-taking behavior in Appendix C (Fig B in S1 Text).

**Fig 6:**
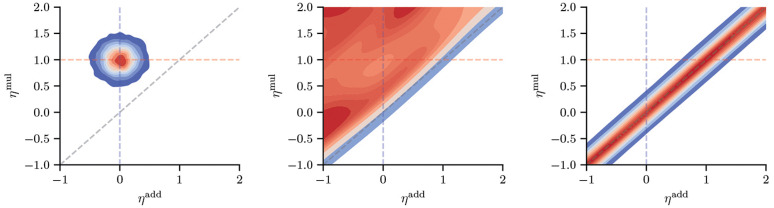
Predicted risk aversion parameter values under the EE, weak EE and EUT model. Risk aversion parameter estimates in the multiplicative session (vertical axis) plotted against estimates in the additive session (horizontal axis). Blue and red dashed lines indicate the theoretical values of risk aversion parameters predicted by the EE model for additive and multiplicative conditions, respectively. The grey dashed line indicates the theoretical values of the risk aversion parameters predicted by the EUT model. **Left,** predictions under the EE model. This predicts that risk aversion parameters cluster around the values that are optimal for growing wealth over time, which is 0 for additive and 1 for multiplicative dynamics. **Middle,** predictions under the weak EE model. This predicts simply that risk aversion parameters are higher under the multiplicative condition than the additive. **Right,** predictions under the EUT model. This predicts participants’ risk aversion parameters are approximately equal under both conditions.

**EE model.** In our experimental setup, two values of the risk aversion parameter play a special role with clear interpretations in terms of time average growth rates. At any current wealth, *x*(*t*), prevailing before a gamble is chosen, an agent following the EE model will optimize the change in the expected transformed wealth associated with each gamble, ⟨δf⟩=⟨f(x(t+δt))⟩−f(x(t)). If we think of one decision trial as a time step δt, the time average growth rate under additive dynamics is [x(t+δt)−x(t)]/δt, and this is δf/δt when η=0. An agent optimising eq. 4 with η=0, therefore, optimises the time average growth rate under additive dynamics. Similarly, the time average growth rate under multiplicative dynamics is [ln(x(t+δt))−ln(x(t))]/δt, and this is δf/δt when the risk aversion parameter approaches 1. An agent optimising eq. 4 with η=1 thus optimises the time average growth rate of wealth under multiplicative dynamics. Agents optimising eq. 4 with any other parameters will, with certainty, experience a slower growth of their wealth in the long run. For instance, an agent with η=0 under multiplicative dynamics will experience slower wealth growth than one with η=1 [5–7]. In settings where dynamics change between additive and multiplicative (as contrived in this experiment), agents that prefer their wealth to grow faster over time must, therefore, adjust their risk aversion parameter between the two conditions from 0 under additive dynamics to 1 under multiplicative dynamics. This predicts a clustering of risk aversion parameter estimates around the (0,1) coordinate as illustrated in Fig 6a.

**Weak EE model.** Several aspects within the experimental setup suggest that it is plausible that human participants will deviate from “optimality” due to the experimental setup rather than because of their actual risk preferences. Notable among these reasons are the relatively short timeframe, the absence of financial losses (in terms of real money payout), and the introduction of wealth control mechanisms. To address these limitations, we also test a weaker version of the EE model, which we refer to as the “weak EE model.” This model, while qualitatively in agreement with the EE model, is not necessarily in quantitative agreement. Specifically, it simply predicts that agents will have a higher risk aversion parameter estimate in the multiplicative condition than in the additive. This predicts a clustering of parameter estimates above the diagonal as illustrated in Fig 6b.

**EUT model.** The framework of using an isoelastic function (eq. 4) does not, by itself, dictate any particular value for the risk aversion parameter. The EE model predicts such values. However, an alternative perspective is that the long term is not relevant to decision-makers and that behaviour is instead dominated by personality and is well described by a single fitted value of the risk aversion parameter, which is person-specific and independent of dynamics. In this model, η is free to vary across participants, but for a given participant, it is the same for both conditions, $\eta^{\text{add}}=\eta^{\text{mul}}$. This predicts a clustering of risk aversion parameter estimates around the diagonal as illustrated in Fig 6c.

### Analysis

**Preprocessing of data.** The raw data are sequences of growth increments/wealth changes associated with images in the gamble pairs and the choices (left or right) made by the participants, which did not require preprocessing. However, trials without a response, as well as trials where either the current wealth or potential outcomes would lead to negative wealth, were excluded. This is because the modelling framework cannot account for negative wealth. The impact of this is minimal due to the experimental design minimising the chances of reaching negative wealth values.

**Data pooling.** For a given dynamic, we carry out the analysis separately and independently for each individual (“no pooling”), producing individual-level parameter estimates. As expected, the choice data from each individual is noisy; therefore, we also produce group-level parameter estimates (“full pooling”). This effectively treats all choices as if a single super-individual made them. Lastly, hierarchical Bayesian modelling (see below) also allows us to model something in between (“partial pooling” [29]). Here, group-level hyperparameters are estimated conjointly with individual-level parameters, with both levels informing each other.

**Parameter estimation.** To estimate the risk aversion parameter introduced in eq. 4, we use Bayesian cognitive modeling [30]. Here, we assume that the probability of choosing the left gamble ($\gamma_{L}$) is related to the difference between the expected changes in transformed wealth associated with the left ($\gamma_{L}$) and right gamble ($\gamma_{R}$). Specifically, we model the probability of choosing the left gamble as the following logistic function.


$$\begin{array}{c} \theta \left(\Delta \left\langle \delta f_{\eta }\right\rangle \right)=\frac{1}{1+e^{-\beta \Delta \left\langle \delta f_{\eta }\right\rangle }}, \end{array}$$ (5)

where $\Delta \left\langle \delta f\right\rangle =\left\langle \delta f_{\eta }(\gamma_{L})\right\rangle -\left\langle \delta f_{\eta }(\gamma_{R})\right\rangle$ and the parameter β controls how sensitive an agent is to differences in expected transformed wealth changes, Δ⟨δf⟩. In the limit, β→∞, agents will always choose the gamble with greater expected transformed wealth.

**Model estimation.** We fit the parameters in eq. 5 via Markov chain Monte Carlo (MCMC) sampling, performed via JAGS (v4.3.0), using MATJAGS (v1.3, psiexp.ss.uci.edu/research/programs_data/jags), interface for MATLAB (2022b). We use 10^4^ samples per chain and four independent chains, with a burn-in of 10^3^ samples per chain. We choose weakly informed priors on η and β. Explicitly, we choose η to be normally distributed with a uniform distribution over the mean and variance ($\mu^{\eta }\sim U(-4,5)$, $\sigma^{\eta }\sim U(0.01,\sqrt{2})$, resulting in a prior spanning a greater range of parameter values than typically empirically observed [9,14], and β to be log-normal distributed, also with a uniform distribution over the mean and variance ($\mu^{\beta }\sim U(-6,8)$, $\sigma^{\beta }\sim U(0.01,\sqrt{2})$, providing a heavy-tailed distribution to allow for the possibility of needing larger sensitivities (Appendix C, Fig G in S1 Text). We checked the convergence of the sampling method by ensuring the Gelman-Rubin R-hat [31] statistic falls between 1 and 1.01, as well as visual inspections of the chain plots (Appendix C, Fig F in S1 Text). In Appendix C (Figs C and D in S1 Text), we further present a different estimation method, which we refer to as the “bracketing method.”

**Statistical tests.** We compute Bayes factors for the effect size, *BF*_10_, via one-sided Bayesian paired *t*-tests using the maximum a posteriori (MAP) estimates of the individual-level risk aversion parameter from each condition, $\hat{\eta }=({\hat{\eta }}^{\text{add}},{\hat{\eta }}^{\text{mul}})$. For this, we use the BayesFactor package (version 0.9.12) implemented in R [32, version 4.3.1], which calculates the Bayes factor on the effect size (ξ). We use the default prior distribution of a half Cauchy with a scale parameter of $\sqrt{2}/2$ and test robustness to prior width over wide (scale 1.0) and ultrawide (scale $\sqrt{2}$) priors. Cohen’s d and Pearson correlations are calculated in R using the lsr package [33]. For exploratory regression analysis, we used the BAS package for R [34] and exported results tables using [35]. All Bayesian credibility intervals are reported as central 95% estimates.

**Hierarchical latent mixture model.** We further set up a hierarchical latent mixture (HLM) model where, by introducing model indicator variables, we can compare qualitatively different behavioural models [30]. We set the priors on η according to the model specifications presented above. Specifically we specify $\eta^{\text{add}}\sim 𝒩(0,\sigma^{\eta })$, $\eta^{\text{mul}}\sim 𝒩(1,\sigma^{\eta })$ for the EE model, $\eta^{\text{add}}\sim 𝒩(\mu^{\eta },\sigma^{\eta })$, $\eta^{\text{mul}}=\eta^{\text{add}}+\delta \eta$, with $\delta \eta \sim \Gamma (2,\frac{1}{2})$ for the weak EE model, and $\eta^{\text{add}}=\eta^{\text{mul}}\sim 𝒩(\mu^{\eta },\sigma^{\eta })$ for the EUT model, with $\mu^{\eta }$, $\sigma^{\eta }$ and β all distributed as specified in the parameter estimation section above. We use a uniform prior over the submodels for the model indicator variable z~Categorical(1/n,…,1/n), where *n* indicates the number of submodels. Note that we run HLM models only containing the specific submodels we are comparing.

### Data and code availability

**Open science framework.** All materials necessary for replicating and reproducing this study are accessible on OSF.io: https://osf.io/mwe7k/. The data has been fully anonymized and can be found within the data component along with precomputed analysis results. The experimental paradigm and analysis code are located in the code component. A short demo video of the paradigm is available at: https://www.youtube.com/watch?v=Isdq_XTQV9I.

**Github.** The codebases are also released on GitHub: https://github.com/ergEx/experiment/releases/tag/v0.5.4 and https://github.com/ergEx/analysis/tree/v1.0.0.

**Pre-data collection registration.** All code, pilot data, and a previous version of this manuscript were preregistered on OSF.io before data collection; see [18].

## Results

**Fig 7:**
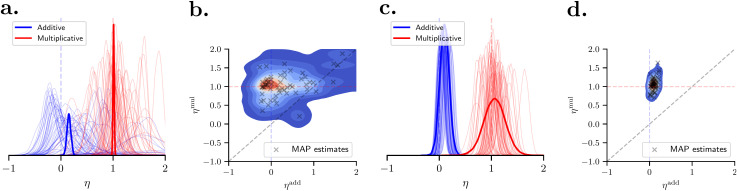
Risk aversion parameter estimates. **a,** the posterior distributions of the risk aversion parameters for additive (blue) and multiplicative (red) conditions. Transparent lines are individual-level estimates using no pooling, and solid lines are group-level estimates using full pooling. **b,** the joint distribution of the risk aversion parameter estimates in the multiplicative and additive conditions. Blue and red dashed lines indicate the values predicted by the EE model for each condition. Grey dashed line indicates the predictions of the EUT model. X marks the maximum a posteriori (MAP) estimate for each individual’s parameter estimation (no pooling). **c,** same as in panel a, but where transparent lines are individual-level estimates, and solid lines are group-level estimates, both derived from the partial pooling method. **d,** same as in panel b, but for the partial pooling method.

**Visualising risk aversion.** We estimated risk aversion parameters independently for each experimental condition. The posterior distributions of the risk aversion parameter, both on the group (full pooling), individual level (no pooling) (Fig 7a), and individual and group parameters with partial pooling (Fig 7c), show a visible increase in the risk aversion parameter estimates in the multiplicative condition. Plotting the joint distribution of these risk aversion parameters for both conditions (no pooling), the central tendency is visibly away from the diagonal line. If the risk aversion parameter were unchanged by the condition, the central tendency would be close to the diagonal (Fig 7b). The same result is visible in the partial pooling, with a tighter distribution due to the hierarchical structure of the model (Fig 7d).

**Data pooling.** To assess which data pooling method best explains the data, we set up an HLM model with three different submodels comprising the different pooling methods: no pooling, full pooling, and partial pooling. To compare these, we compute PXP to quantify the probability of being the most frequent in the population, revealing partial pooling as the most frequent model (PXP no pooling = $6.001\times 10^{-11}$, PXP partial pooling ≈ 1, PXP full pooling = $6.001\times 10^{-11}$; Appendix D, Fig H in S1 Text). This corresponds to an extreme level of evidence in favour of the partial pooling model compared to the two other models (full pooling BF = $2.749\times 10^{121}$, no pooling BF = $2.537\times 10^{158}$; see Fig J in S1 Text for individual-level Bayes factors). In line with our pre-registration [18], and formally justified by this model comparison, we focus subsequent analysis primarily on the parameter estimates from the partial pooling method.

**Fig 8:**
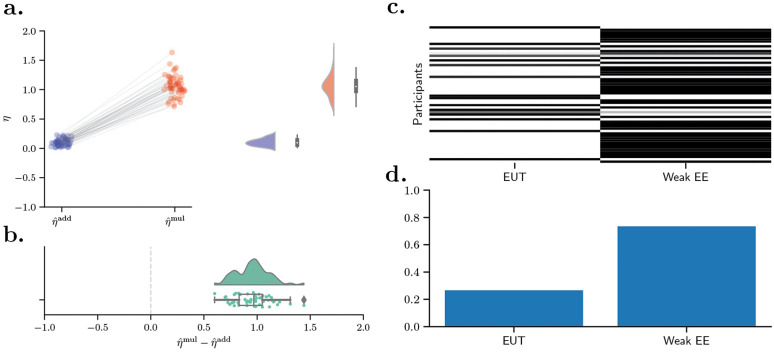
Comparison between the weak EE model and the EUT model. a, raincloud plot of the risk-aversion parameters, estimated by the partial pooling method. Lines between points indicate the same participant. **b,** pairwise difference of the parameter estimates in the two conditions. **c,** posterior model probabilities per subject. **d,** the same, marginalized over subjects.

**Risk aversion parameters are higher in the multiplicative condition.** In line with our pre-registration [18], we compare the weak EE model with the EUT model via a one-sided paired Bayesian t-test using maximum a posteriori estimates of the risk aversion parameter based on the partial pooling parameter estimates. Specifically, we test the hypothesis that ${\hat{\eta_{i}}}^{\text{add}}<{\hat{\eta_{i}}}^{\text{mul}}$ against the null hypothesis of ${\hat{\eta_{i}}}^{\text{add}}={\hat{\eta_{i}}}^{\text{mul}}$. This t-test reveals strong evidence in favour of the weak EE model (${\text{BF}}_{\text{EE},\text{EUT}}^{t}>1.707\times 10^{43}$, effect size ξ=5.982, ${\text{BCI}}_{2.5\%}^{\xi }=4.795$, ${\text{BCI}}_{97.5\%}^{\xi }=7.051$). We find that all 58 participants show an increase in the risk aversion parameter from the additive to the multiplicative condition (Fig 8a). This fact is also visible in the pairwise differences for the estimates in the multiplicative session minus the estimates from the additive session (Fig 8b). Finally, the increase in risk aversion parameter under the multiplicative condition is supported by a hierarchical latent mixture model, which indicates strong evidence in favour of the weak EE model (BF = $6.982\times 10^{111}$, ${\text{PXP}}_{\text{EE}}=9.961\times 10^{-1}$, ${\text{PXP}}_{\text{EUT}}=4.177\times 10^{-3}$; Fig 8c; see Fig I in S1 Text for individual-level Bayes factors of weak-EE vs EUT and EE vs EUT under no pooling). The model probabilities can be seen in Fig 8d, where substantively more participants are attributed to the weak EE model than the EUT model.

**Confounders.** In an exploratory analysis to test for confounders, we deployed a Bayesian linear model combined with model averaging, regressing possible confounders on the pairwise difference of risk aversion parameters between the two conditions. This analysis reveals that no other differences (e.g., pairwise differences in luck, variance in wealth, or log wealth) between the conditions can explain the difference in estimated risk aversion parameters. We find that Bayes factors for the inclusion of all factors have moderate to strong evidence against their inclusion (*BF* < 0.115; see Appendix D). This suggests that the dynamical condition is unique in its ability to explain differences in observed risk aversion parameters. As further checks, we tested whether simple trial-to-trial reinforcement heuristics (staying based on side, gamble variance, and gamble variance conditional on a prior high-variance choice) could account for the observed pattern; they do not (Appendix D, Fig K in S1 Text). We also verified that the same shift in risk aversion is present when the analysis is restricted to either the first or the second half of the decision trials, indicating that the effect is not driven by continued learning during the decision phase (Appendix D, Fig M in S1 Text).

**Participants are close to optimizing the time average growth rate.** To test the quantitative predictions of the EE model, we compare it directly with the EUT model. Again, we use the maximum a posteriori estimates from the risk aversion parameter distributions under the partial pooling method. To do this, we first consider the estimates $\hat{\eta }=({\hat{\eta }}^{\text{add}},{\hat{\eta }}^{\text{mul}})$ in the $\left(\eta^{\text{add}},\eta^{\text{mul}}\right)$-plane for each participant and then measure its Euclidean distance from the optimal time average growth rate coordinate (0,1) (*d*_EE_) and compare this with the equivalent distance from the diagonal (*d*_EUT_). In other words, we calculate how far the estimated risk aversion parameter is from the prediction of the EE model and how this compares to how far it is from the prediction of the EUT model.


$$\begin{array}{c} d_{\text{EE}}=d(\hat{\eta },(0,1))=\sqrt{({\hat{\eta }}^{\text{add}}-0{)}^{2}+({\hat{\eta }}^{\text{mul}}-1{)}^{2}}, \end{array}$$

$$\begin{array}{c} d_{\text{EUT}}=d(\hat{\eta },(x,x))=\sqrt{({\hat{\eta }}^{\text{add}}-x{)}^{2}+({\hat{\eta }}^{\text{mul}}-x{)}^{2}}\;=\;\frac{\left|{\hat{\eta }}^{\text{add}}-{\hat{\eta }}^{\text{mul}}\right|}{\sqrt{2}}. \end{array}$$

**Fig 9:**
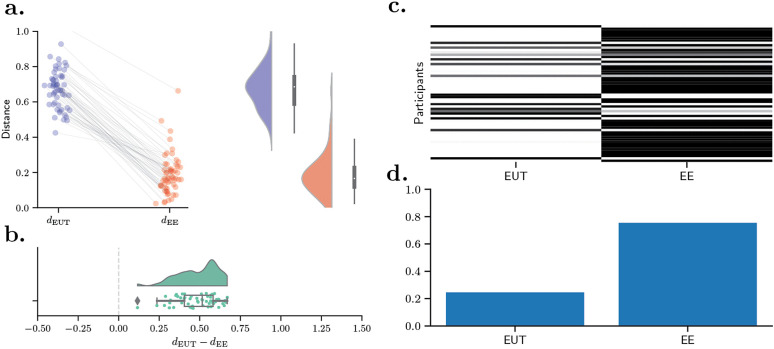
Comparison between the EE model and the EUT model. **a**, raincloud plot of the distance between the risk-aversion parameters estimated by the partial pooling method and the predictions of the EUT model and the EE model, respectively. Lines between points indicate the same participant. **b,** pairwise difference of the distances. **c,** posterior model probabilities per subject. **d,** the same, marginalized over subjects.

The distance estimates for each model are presented in Fig 9a and their pairwise differences in Fig 9b. Using a one-sided Bayesian paired t-test, we compare the two distance measures. Specifically we test the hypothesis that $d_{\text{EUT}}>d_{\text{EE}}$ against the null hypothesis that $d_{\text{EUT}}\leq d_{\text{EE}}$. This revealed extreme evidence in favour of the EE model (${\text{BF}}_{\text{EE},\text{EUT}}^{t}>1.999\times 10^{36}$, estimated effect size ξ=3.960, ${\text{BCI}}_{2.5\%}^{\xi }=3.146$, ${\text{BCI}}_{97.5\%}^{\xi }=4.689$). To support this, we embedded the EE and EUT models as submodels in a hierarchical latent mixture model. Bayesian model comparison between these two submodels reveals extreme evidence in favour of the EE model (BF = $4.014\times 10^{121}$, ${\text{PXP}}_{\text{EE}}=9.994\times 10^{-1}$, ${\text{PXP}}_{\text{EUT}}=5.683\times 10^{-4}$; Fig 9c&d).

**Fig 10:**
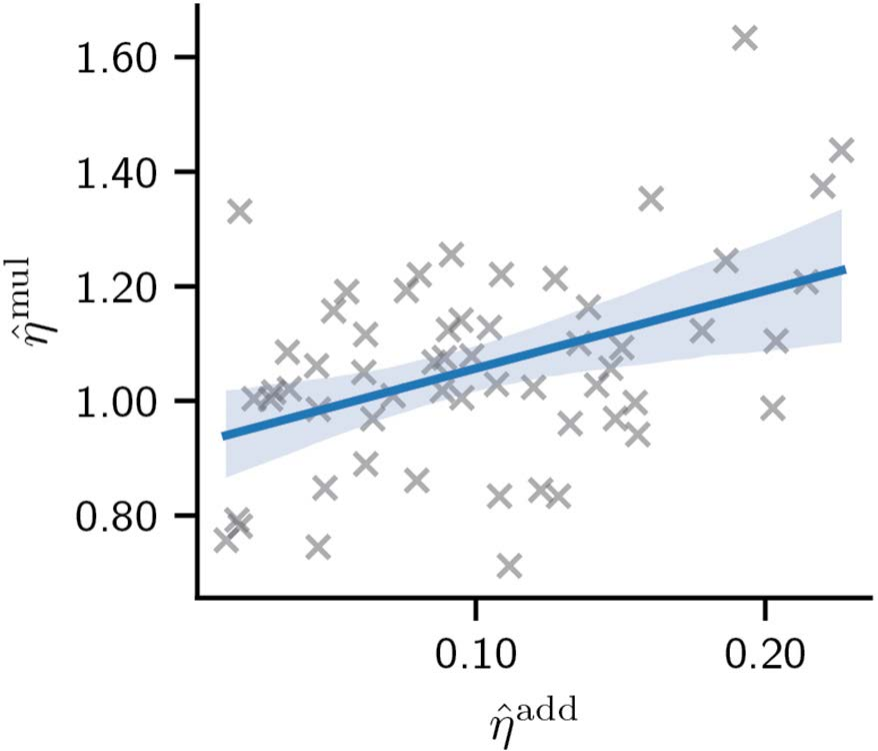
Bayesian correlation. Correlation plot of the maximum a posteriori estimate of the risk aversion parameter in the multiplicative condition versus additive (one cross for each participant). The blue line is the best-fit linear relation, and the light blue shaded area indicates its 95% bootstrap confidence interval.

**Trait-like risk preferences persist across conditions.** To test for trait-like risk preferences within participants, we ran a Bayesian correlation test (Pearson’s ρ) between the maximum a posteriori estimates of the risk aversion parameter between the two conditions. We find extreme evidence for a positive correlation (${\text{BF}}_{+,0}\approx 139$, ρ=0.440, ${\text{BCI}}_{2.5\%}^{\rho }=0.210$, ${\text{BCI}}_{97.5\%}^{\rho }=0.626$, Fig 10). This indicates that we are capable of capturing individual differences consistent with trait-like risk preferences. However, the fact that the difference in risk aversion parameters between the dynamical conditions is nearly 6 times larger than the standard deviation of the within-participant differences (i.e., effect size ξ=5.982) indicates that despite the detectable trait variation over participants, the dynamic is the dominant source of variation.

**Bayesian linear regression on risk aversion parameters.** In an exploratory analysis to evaluate the dependency of the condition-specific risk aversion parameters on other variables, including established risk questionnaires, we performed a Bayesian linear regression with Bayesian model averaging as implemented in the BAS package for R. We first do this with the additive risk aversion parameter estimates as the dependent variable. With independent variables being sex, income, age, DOSPERT sub-scales of each of the three measures, the score of the risk-propensity inventory, and the risk-aversion estimates of the multiplicative risk-aversion estimates and the risk aversion estimate from the multiplicative condition. Averaged over all possible model combinations that include or exclude a given variable, the only inclusion Bayes factor for the risk aversion parameter from the multiplicative condition has even moderate evidence (${\text{P}}_{inclusion}=0.895$, ${\text{BF}}_{\text{inclusion}}\approx 8.501$; see Appendix D). We performed the same analysis but with the multiplicative risk aversion parameter as the dependent variable. Equivalently, the only factor with moderate evidence is the additive risk aversion parameter (${\text{P}}_{inclusion}=0.880$, ${\text{BF}}_{\text{inclusion}}\approx 7.320$; see Appendix D).

**Fig 11:**
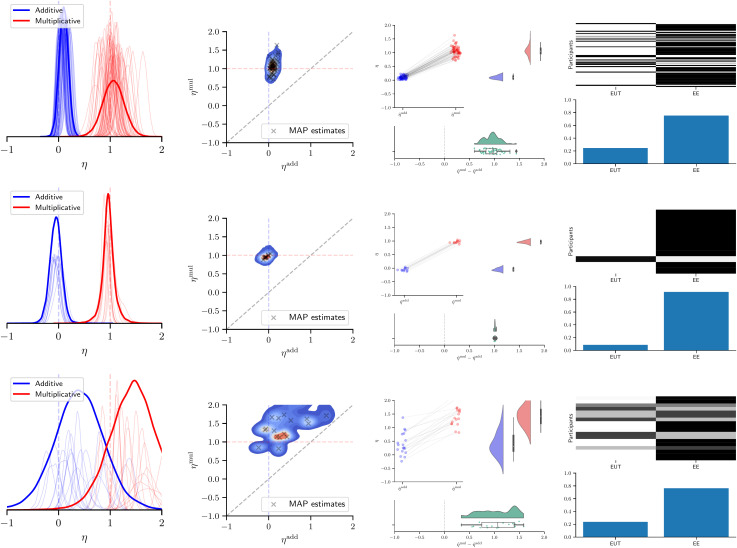
Replication and generalisation of results over different datasets. Different rows represent results from different data sets, from top to bottom: current dataset, pilot dataset, Meder dataset. The columns represent different visualisations of the results. **First column,** the posterior distributions of the risk aversion parameters for additive (blue) and multiplicative (red) conditions. **Second column,** the joint distribution of the risk aversion parameter estimates. **Third column,** raincloud plot of the maximum a posteriori estimates and pairwise difference in the two conditions. **Fourth column,** posterior model probabilities for the EUT model and the EE model, respectively, based on the model indicator variable.

**Replication.** To assess the degree to which we can replicate the results presented here, we compare our results with the results from two other datasets. We refer to the data presented above as the “Current dataset,” the data presented in [14] as the “Meder dataset” and the data presented in [18] as the “Pilot dataset.” The current and pilot datasets were generated from identical protocols and experimental designs, whereas the Meder dataset was generated using a similar experimental design but with different protocols that had some key differences (e.g., wealth being invisible and not updated in real-time, and restrictions on the trajectories in the learning task). As such, the pilot and current datasets can be seen as a generalisation of [14]. In the Meder dataset, participants are generally biased to be more risk averse than predicted by the EE model; however, in the pilot and current datasets, participants’ risk aversion parameters centre closely on the optimal values of 0 and 1 (Fig 11, 1st column) for which wealth grows fastest over time. The analysis code for all three datasets was the same. In all three datasets, the bivariate location of the risk aversion parameter is approximately the same (Fig 11, 2nd column). In all three datasets, the pairwise differences show consistent increases in risk aversion parameter in the multiplicative condition, and not just on the group average but also for the individual estimates, and pairwise differences are all quantitatively similar (Fig 11, 3rd column). In all three datasets, the posterior model probabilities for the participant predominantly favour the EE model over the EUT model (Fig 11, 4th column). Finally, in all three datasets a Bayesian model comparison between an EE model and the EUT model, the group Bayes factors (GBF) indicate extreme evidence in favour of the EE model (current dataset, GBF = $4.014\times 10^{121}$, PXP = 0.999), pilot dataset (GBF = $2.611\times 10^{40}$, PXP = 0.982), and Meder dataset (GBF = $4.564\times 10^{37}$, PXP = 0.986).

## Discussion

We conducted an experiment where participants made consequential risky decisions under additive and multiplicative gamble dynamics. In our pre-registered analyses, we find convergent and strong evidence that the participants’ behaviour was well predicted by time average growth rate maximization. This is evident in the risk aversion parameter estimates centring on 0 in the additive condition and 1 in the multiplicative condition. Further analysis also reveals that once dynamics are accounted for, trait-like differences are observed. This highlights that both dynamics and traits are necessary components in a complete theory of risk-taking behaviour, but in this artificial setting we find that dynamics are the dominant source of variation.

The current experiment was designed in the context of having previously performed a similar experiment in 18 participants [14]. There, it was found that the risk aversion parameter increased in the multiplicative condition compared to the additive condition and that the parameter values were approximately what is quantitatively predicted by time average growth rate maximisation (Fig 11, lower row). In the present experiment, we aimed to replicate and generalise these results by addressing criticisms of the original experiment [14]. Firstly, in the original experiment, wealth and gamble outcomes were hidden from the participants. This opened the possibility of an uncontrolled effect where participants guessed their current wealth, potentially influencing their behaviour. Secondly, the final payout for participants was computed via the realisation of only a subset of choices. In the present experiment, all decisions were immediately consequential to the participant’s in-game wealth, and the payouts were proportional to the final in-game wealth. Lastly, the wealth trajectories shown to participants in the learning task were bounded and controlled, which led to systematic differences between the conditions in terms of what was experienced. In the present experiment, we removed these boundaries and randomly sampled from the available stimuli, thus eliminating the possibility that this caused the results. The observed effect is unlikely to depend solely on the presence of an explicit wealth display: in our earlier study [14], wealth and gamble outcomes were not displayed, yet we observed quantitatively similar adaptation of the inferred transformation across dynamics (Fig 11, lower row). This suggests that omitting the wealth display would not eliminate the core effect.

Although these considerations cannot explain how the participant’s risk aversion parameter estimates quantitatively match the growth-optimal strategy, the new design nevertheless eliminates them as possible confounders. Despite the differences in the design—different stimuli, a different mapping from in-game wealth to real monetary payouts, different wealth visibility, different learning tasks, and half the number of trials— we replicate the results under the new design (Fig 11). As predicted by time average growth rate maximization, the risk aversion parameter increases under the multiplicative condition compared to the additive.

Strictly speaking, time average growth rate maximization predicts that participants should have a risk aversion parameter (eq. 4) of exactly 0 in the additive condition and 1 in the multiplicative. In the multiplicative condition, we find that the posterior estimates for the group are almost exactly as predicted (mode = 1.062, *BCI*_95%_ [0.655, 1.463]). The wealth transformation with this parameter value is almost exactly the logarithmic function. Note that this has an interesting correspondence to the widely used Kelly criterion [13], which for settings where bets are also made under multiplicative dynamics, is an optimal strategy that maximises the expected value of the logarithm of wealth. In other words, agents that behave according to the Kelly criterion are performing the same logarithmic wealth transformation as we have empirically resolved in this experiment. In the additive condition, we find that the posterior estimates for the group are slightly higher than predicted (mode = $0.0987,BCI_{95\%}[-0.0752,0.2710]$). The same positive offset was observed in [14]. While we do not have empirical evidence as to why this is the case, we speculate that this might, in part, be due to the experimental setup. In contrast to many real-life situations, where a participant, for example, with zero or negative wealth, will face different dynamics (such as interest or default), the participant’s ability to win or lose in this task is independent of their wealth level. It is thus not too surprising that participants might not fully adapt to this condition and behave with slightly greater risk aversion than predicted.

As with all laboratory experiments—using idealised and controlled conditions—the question arises of how our findings generalise to the real world. In the context of our replications across different samples and despite having excluded participants with formal quantitative training, we find that human participants are capable (given the right conditions) of learning and making decisions according to time average growth rate maximisation. Such cognition is thus at least possible and detectable in all participants we have sampled to date. This speaks to the plausibility of observing such behaviour outside the laboratory.

An important aspect of our findings is the timescale on which the change in risk aversion parameter takes place. A common narrative pertains to human utility functions being formed over evolutionary timescales and possibly anchored to hard-wired sensory mechanisms [36]. Our experimental results pose a challenge to this narrative: Utility functions or simply wealth transformations can adapt substantively in response to dynamics on timescales of minutes, not millennia. This suggests that evolution has not favoured a specific set of utility functions but rather a mental agility which allows people quickly to adapt such wealth transformations to the dynamics of their environment. Whether this is true for other than additive and multiplicative dynamics remains an open question.

While our central finding is that dynamics exert a strong and systematic effect, we also find systematic within-person biases. The data presented here thus suggests dynamics is a dominant factor when explaining risk-taking behaviour but does not eliminate the notion of individual idiosyncratic preferences. This is in accordance with a recent study that found that only about 15% of the variance in risky decision-making could be explained by demographic and personality scores [37]. Specifically, we see this in our data. The more risk-averse (i.e., the participants with higher risk aversion parameters) in the additive condition are also among the more risk-averse in the multiplicative condition. This speaks to the importance of disentangling trait-like and environmentally-driven variation. If this is done correctly, the notion of an individual being “risk-averse” or “risk-seeking” can be meaningful.

Our data suggest that humans adapt their risk-taking behaviour on very short timescales and appear to do so in line with the principle that participants are trying to maximize the time average growth rate, and they have an intuitive understanding of how to do this. The dominance of expected utility theory has led to a general mindset in the field where dynamical effects are often ignored, and their practical importance is underestimated. The present experiment was designed to uncover dynamical effects. It yields a clear result: dynamics affect decision-making and can dominate over idiosyncratic preferences. This experimental result is in line with emerging evidence from large-scale panel data, where risk attitudes have been found to change in response to environmental fluctuations over timescales of months [38]. Together, our experimental result replicates and generalises the finding that risk-taking behaviour can be explained by a principle of time average growth rate maximization and further motivates the need to develop and test theories of decision-making that are explicitly founded on ergodic considerations.

## Supporting information

S1 TextCombined Supporting Information document containing Appendices A–E, Tables A–D, and Figs A–M.These components are provided together in the single Supporting Information file; their captions are listed below. **Table A. Regression of potential confounding variables on the pairwise difference in risk aversion parameters between dynamics.** Bayesian linear model with model averaging using as predictors pairwise differences (multiplicative minus additive) in luck, terminal wealth, variance of log-wealth, and variance of wealth. For each predictor we report the posterior inclusion probability P(β≠0∣Y), posterior mean, posterior SD, and inclusion Bayes factor ${BF}_{incl}$. No predictor reaches substantive evidence for inclusion. **Table B. Bayesian linear regression on the additive-condition risk aversion parameter (**$\eta_{add}$**).** Predictors include the multiplicative-condition risk aversion parameter (partial-pooling estimate); the Risk-Taking, Risk Perception, and Expected Benefits sub-scales of the 30-item DOSPERT, evaluated across its five risk domains (Ethical, Financial, Health/Safety, Recreational, Social), with the Financial domain further split into Gambling and Investment sub-scales (yielding six codes per sub-scale); the Risk Propensity Scale (RPS); age; sex; and income. Columns as in Table A. **Table C. Bayesian linear regression on the multiplicative-condition risk aversion parameter (**$\eta_{mul}$**).** Identical predictor set to Table B except that the additive-condition risk aversion parameter (partial-pooling estimate) is substituted for the multiplicative one as a predictor. Columns as in Table A. **Table D. Bayesian linear regression on the within-participant change in risk aversion parameter (**$\Delta \eta =\eta_{mul}-\eta_{add}$**).** Predictors include the DOSPERT sub-scales, RPS, age, sex, and income. Columns as in Table A. **Fig A. Bayes factor design analysis. a,** Sequential designs under a zero effect size. **b,** Sequential designs under a medium effect size (Cohen’s *d* = 0.5). **Fig B. Visual representation of the isoelastic utility function.** Two qualitatively different regimes are illustrated: η<0 (convex) and η>0 (concave). The same gamble (stay at 500 vs. a fair coin toss winning/losing 300) is evaluated in each case to show how the sign of η determines the choice. **Fig C. Construction of the bracketing estimation method. Left,** a gamble choice implies either a lower or upper bound on a participant’s risk aversion parameter at the gamble pair’s indifference η. **Middle,** plotting lower bounds at 0 and upper bounds at 1 yields a step function in the noiseless limit, with the participant’s true η at the step. **Right,** fitting a logistic curve to the bounds gives a point estimate via its inflection point. **Fig D. Risk aversion parameter estimates from the bracketing method. a,** Posterior distributions of η for additive (blue) and multiplicative (red) conditions; transparent lines, individual-level; solid lines, group-level. **b,** Joint distribution of individual MAP estimates across conditions; blue/red dashed lines indicate EE-model predictions; grey dashed line indicates the EUT-model prediction. **Fig E. Parameter recovery from synthetic agents.** Individual risk aversion parameter estimates recovered for synthetic agents with stable ground-truth values {(−0.5,−0.5),(0,0),(0.5,0.5),(1,1),(1.5,1.5)} (consistent with the EUT model) and one EE-consistent value (0,1). **Left,** Bayesian partial pooling. **Middle,** Bayesian no pooling. **Right,** bracketing method (no pooling). **Fig F. MCMC sampling traces. Left,** traces of risk aversion parameters (additive and multiplicative) for the no-pooling model. **Right,** the same for the partial-pooling model. **Fig G. Estimates of the sensitivity parameter** β**. Left,** posterior estimates of logβ for the no-pooling model (transparent: individuals; solid: group, full pooling). **Right,** the same for the partial-pooling model. **Fig H. Data pooling comparison. Upper,** posterior model probabilities for each pooling model based on the model-indicator variable. **Lower,** the same summed over participants. **Fig I. Bayes factor analysis (no-pooling data). Left,** individual log Bayes factors for weak EE vs. EUT. **Right,** individual log Bayes factors for EE vs. EUT. **Fig J. Bayes factor analysis (partial-pooling data). Left,** individual log Bayes factors, partial pooling vs. no pooling. **Right,** individual log Bayes factors, partial pooling vs. full pooling. **Fig K. Paired differences in stay probabilities by previous-trial reinforcement.** Strip plots of paired differences in logit-transformed stay probabilities between rewarded and non-rewarded previous trials, with paired-*t* statistics (mean and 95% CI). Three features per session are tested: same side; same gamble variance; same gamble variance conditional on having chosen the higher-variance gamble on the previous trial. The right panel zooms out the high-variance comparison. **Fig L. Difference in no-brainer accuracy between conditions.** Per-participant difference in proportion-correct (additive minus multiplicative), shown by run and averaged across runs. Five-number summaries for the group are shown with dots indicating individual performance. **Fig M. Risk aversion parameters estimated from each half of the decision phase.** Joint distribution of posterior MAP estimates for additive and multiplicative conditions, computed separately from the first half (left) and second half (right) of decision trials. Fiducials indicate the time-optimal predictions (blue: additive; red: multiplicative) and the dynamics-invariant EUT prediction (grey diagonal).(PDF)
