# Supplementary material for: Ergodicity transformations predict human decision-making under risk
Source: PLoS Comput Biol. 2026 Jul 20;22(7):e1014409. doi: 10.1371/journal.pcbi.1014409 (PMC13472505; doi:10.1371/journal.pcbi.1014409)
Supplement: Supplementary file 1 [file pcbi.1014409.s001.pdf]

# Supporting Information: Ergodicity transformations predict human decision-making under risk

BENJAMIN SKJOLD, SIMON R. STEINKAMP, COLM CONNAUGHTON, OLE PETERS, OLIVER J. HULME

## ABSTRACT

This document contains the Supporting Information for [1] / the PLOS Computational Biology submission *Ergodicity transformations predict human decision-making under risk*. It is the single combined Supporting Information item, **S1 Text**. Figures and tables are labelled alphabetically (Fig A, Fig B, ...; Table A, Table B, ...) and are referred to as such from the main text (e.g. "Fig A in S1 Text").

## Appendix A: Participants

**Performance-related payoffs.** The payoff of each participant in each of the two sessions was calculated by creating a prize pool of DKK 2500 (approx. USD 365); see prize pool specification below. For each session, the prize pool was distributed in proportion to each participant's final points. For example, a participant who obtained 10% of the points of their pool earned 10% of the prize pool. Negative final points were clipped to zero prior to the calculations. This procedure was repeated independently for each session (note participants were compared within dynamics regardless of which dynamic they were presented with first). To avoid gambling for "peanuts" [2], where risk preferences might be different for insignificant amounts, the prize pools were sized to be perceived as substantive amounts of money. Participants were thus incentivised to pay attention to all images and to optimise their learning and decision-making throughout the experiment.

**Prize pools.** We created prize pools by utilizing a rolling window of 10 participants. In this setup, each participant, denoted as $n$, was compared with the preceding 9 participants, namely $(n-1, n-2, \ldots, n-9)$. The first 10 participants comprised a single prize pool. The rolling window provided a fair mechanism for dealing with the experiment stopping with a number of participants not divisible by 10.

**Payouts.** In the additive condition, participants received on average DKK 248.4 ($\sim$ USD 36) with a standard deviation of DKK 164.6 ($\sim$ USD 24), and in the multiplicative condition, on average DKK 285.6 ($\sim$ USD 41) with a standard deviation of DKK 389.5 ($\sim$ USD 56).

## Appendix B: Protocol

**Bayes factor design analysis.** Prior to data collection, we ran a Bayes Factor Design Analysis [3, 4, BFDA] to assess the impact of minimum and maximum participant numbers. The equivalent effect size based on a previous dataset [1, 5] was large. However, we conservatively opted for a medium effect size (Cohen's d, $\xi = 0.5$). This revealed that under this design, for a Bayesian paired t-test, 100% of studies correctly hit the H1 boundary (panel b of Fig A). For a zero effect size, 71.8% of studies correctly hit the H0 boundary (1.6% incorrectly hit the H1 boundary, and 26.6% terminated inconclusively at 150 participants; panel a of Fig A). 90% power, defined as the probability of accepting H1 over H0 given that H1 generated the data (with a medium effect size), is achieved after 69 participants. The upper bound of 150 participants thus exceeds this minimal standard of power. The BFDA was run in R; the code is available in the ANALYSIS folder under R_ANALYSES.

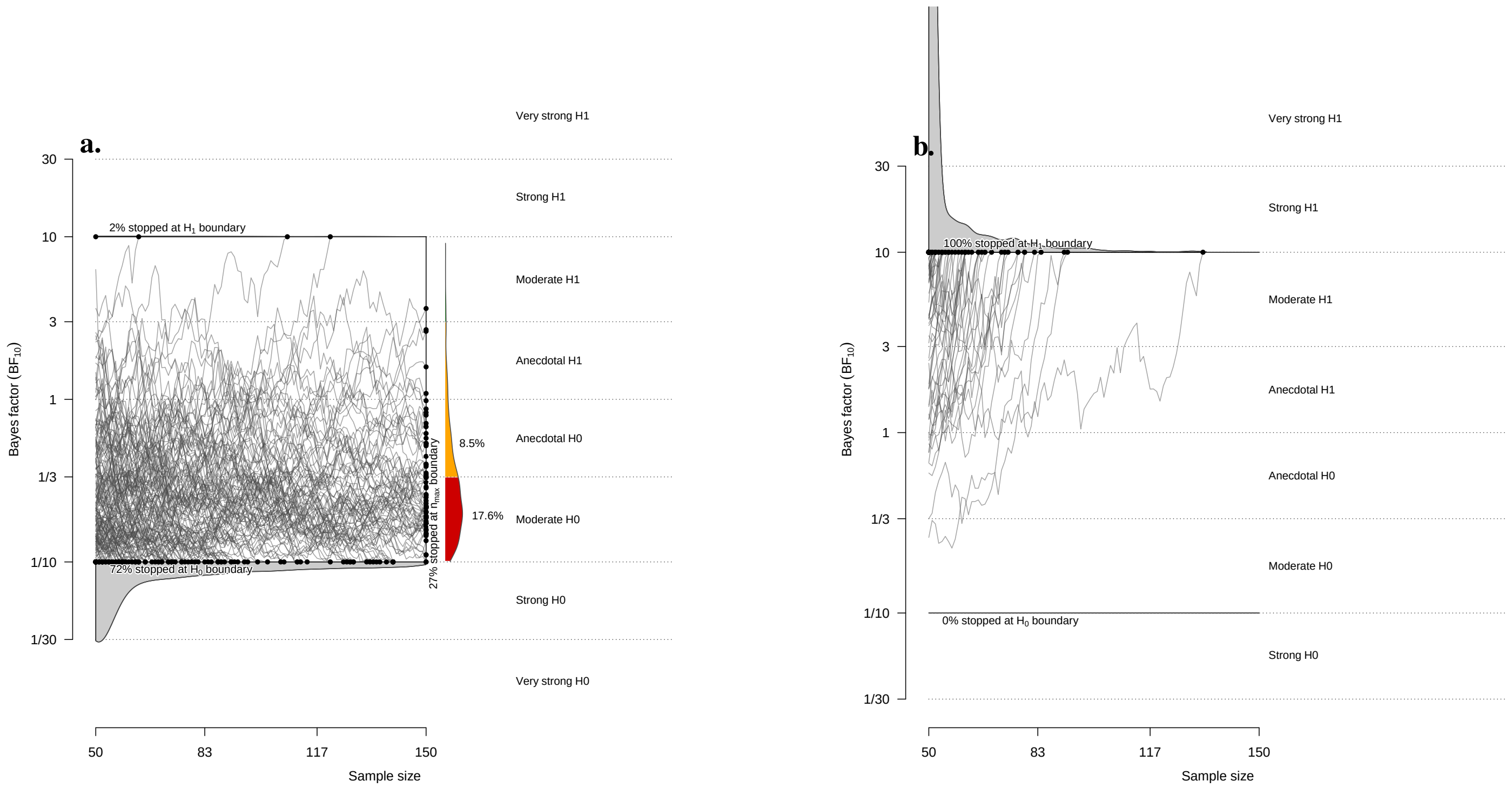


**Fig A. Bayes factor design analysis. a,** Sequential designs for zero effect size. **b,** Sequential designs for a medium effect size (Cohen's d = 0.5).

**Stimulus selection.** We used abstract images from a validated corpus of abstract fractals [6], specifically the subset of 60 that was previously used in a memory task. Of these, we selected those scoring highest in discrimination performance and memorability [6] and removed those that received similar descriptive labels, resulting in a final image bank of 50 images. These images and the code for stimulus selection and resampling can be found in the EXPERIMENT folder under DATA/STIMULI. The selected images were resized to 425 by 319 pixels using a bicubic resampling filter (implemented in Pillow). From this image bank, for each participant, we randomly sampled (without replacement) two sets of nine images each. This way, each participant saw, in each session, a set of nine unique images. Furthermore, this approach ensured that the association between wealth increments and images were randomized across the whole study.

**Wealth control mechanism.** As discussed in [7], there exists a limited wealth range within non-multiplicative conditions in which the chosen family of utility functions (see Eq. 4) allows us to distinguish between agents with different risk aversion parameters. In one way, the issue is that Eq. 4 is only defined for positive values. The ability of an agent to experience negative wealth and bounce back into positive wealth is, however, an important feature of the additive dynamic. We, therefore, set a softbound (described below) at $-500$ but disregard all trials where current wealth is or any possible outcome leads to negative wealth from the analysis. In the positive direction, we see a phenomenon which we refer to as decision convergence, where (at a finite wealth) agents, regardless of their risk aversion parameter, will make the same decisions. Through simulations, we find that around a wealth of 2500, our ability to distinguish between agents with realistic parameter values diminishes. We, therefore, set an upper softbound here. **Softbounds.** It is unknown to what extent such wealth bounds have an effect on participants' choices. We, thus, set them to be soft, meaning that even when reaching the upper bound, it was still possible for the participant to increase their wealth in the hope that participants didn't notice (and thus act) on such an intervention. To be explicit, when a softbound was reached, we removed all gamble pairs containing the two images that would result in the most extreme wealth change in a direction toward or past the bound. The participants could, therefore, still win or lose wealth after reaching one of the soft boundaries. Under these constraints, the gamble pair space is reduced to contain 35 gamble pairs. **Multiplicative condition.** In the multiplicative condition, neither negative wealth nor decision convergence is relevant. However, to avoid confounds between the two conditions, we set similar softbounds in the multiplicative session. These were set by calculating the number of standard deviations to reach the bounds set in the additive session under $\Omega^+$ and then matching these in the multiplicative session under $\Omega^\times$, i.e.

$$x(0) \pm \mathrm{std}(\Omega^+) \times x = x(0) \pm 1,500 \quad \Rightarrow \quad x = 5.438 \tag{1}$$

$$\Rightarrow x(0) \pm \mathrm{std}(\Omega^\times) \times 5.438 = A \quad \Rightarrow \quad A = \begin{cases} 64 \\ 15,589 \end{cases} . \tag{2}$$

We thus get that the lower softbound is set at $-500$ (additive) and 64 (multiplicative) and the upper softbound at $2,500$ (additive) and $15,589$ (multiplicative) given the initial wealth, $x(0) = 1,000$.

## Appendix C: Modelling framework

### Descriptive framework

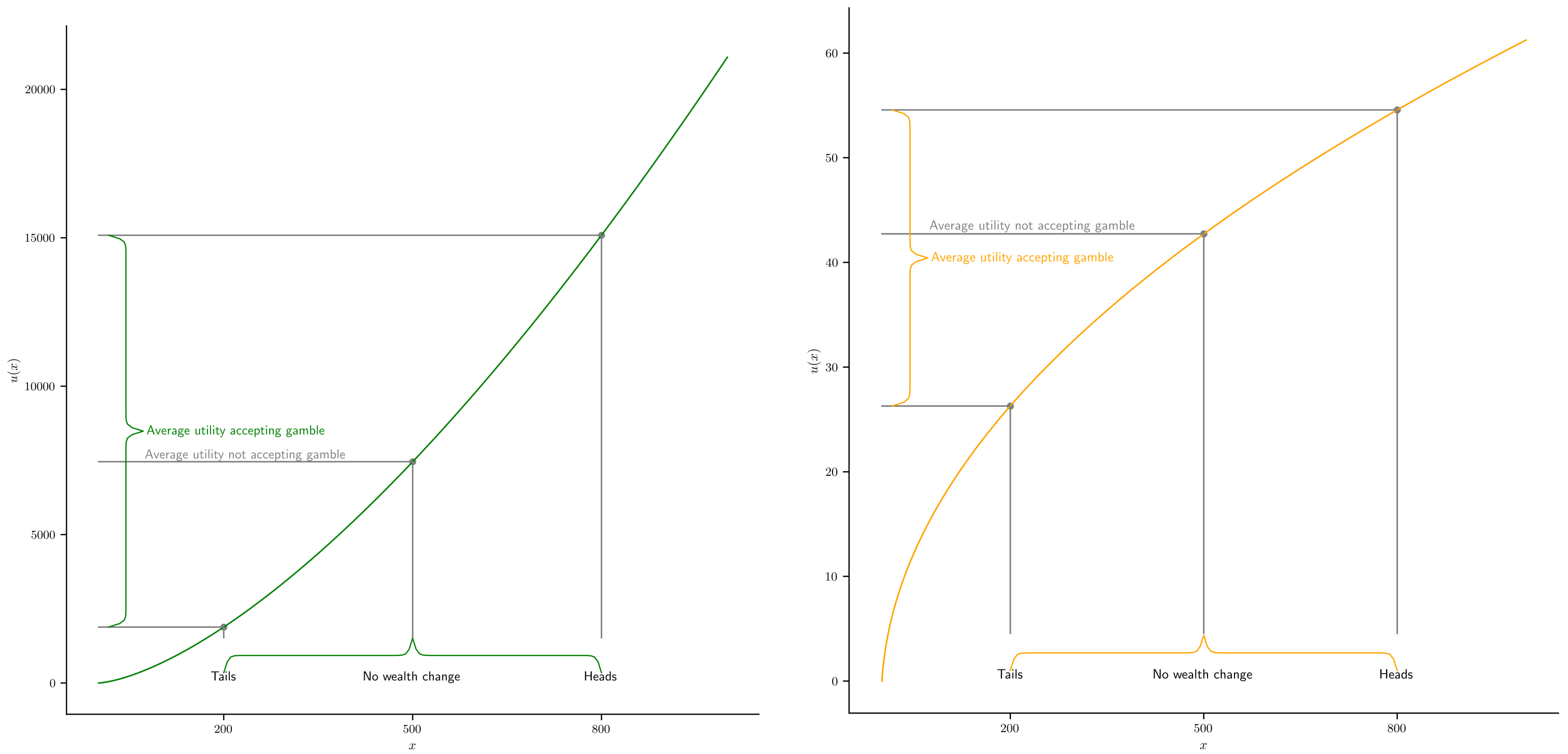


**Fig B. Visual representation of the utility function** Showcasing two qualitatively different regimes for the chosen family of utility functions (Eq. 4), *i.e.* $\eta < 0$ resulting in a concave function and $\eta > 0$ resulting in a convex function. For both, the same choice between staying at 500 (not accepting the gamble) or a coin toss between winning or losing 300 (accepting the gamble) is offered. **Left,** agent with $\eta = -0.5$. The average utility of accepting the gamble is larger than the average utility of not accepting the gamble, and thus, this agent will accept the gamble. **Right,** agent with $\eta = 0.5$. The average utility of accepting the gamble is lower than the average utility of not accepting the gamble, and thus, this agent will not accept the gamble.

### Alternative estimation methods

**Bracketing method.** We further implemented an alternative estimation method to provide an independent validation of the estimated risk aversion parameter. For any given gamble pair, we define the (wealth-dependent) indifference value, $\eta^{\text{ind}}(\zeta, x)$, of the risk aversion parameter. Under the model framework presented in the main paper, this is the risk aversion parameter value at which an agent is indifferent between the two gambles on offer. This is the case when the expected utilities associated with the left and right gambles are identical, meaning the indifference value $\eta^{\text{ind}}$ satisfies the following equation:

$$\underbrace{\frac{1}{2}\left\{u\left[x+\delta x(\omega_{\text{L}}^{\text{top}});\eta^{\text{ind}}\right]+u\left[x+\delta x(\omega_{\text{L}}^{\text{bottom}});\eta^{\text{ind}}\right]\right\}}_{\left\langle \delta u(\gamma_{\text{L}});\eta^{\text{ind}}\right\rangle}=\underbrace{\frac{1}{2}\left\{u\left[x+\delta x(\omega_{\text{R}}^{\text{top}});\eta^{\text{ind}}\right]+u\left[x+\delta x(\omega_{\text{R}}^{\text{bottom}});\eta^{\text{ind}}\right]\right\}}_{\left\langle \delta u(\gamma_{\text{R}});\eta^{\text{ind}}\right\rangle}. \quad (3)$$

Each gamble pair shown in a decision trial is associated with such an indifference value, $\eta^{\text{ind}}(\zeta, x)$. Note: It is possible to construct gamble pairs where no value of $\eta$ satisfies eq. 3. However, all such gamble pairs were excluded from the experiment. When a participant makes a choice (left or right), this reveals that for that specific choice, the participant would be best modelled with a risk aversion parameter value bounded by the trial's indifference value (a similar logic is taken in interval regression [8, 9]). If the higher variance gamble is chosen, then that indifference value is an upper bound, and otherwise, it is a lower bound. A sequence of choices can thus be translated into a sequence of upper and lower bounds on the participant's risk aversion parameter, $\eta$. An ideal agent, namely one acting precisely according to the model framework, will make choices such that all implied lower bounds are below the agent's true risk aversion parameter value and all upper bounds are above it. With at least one upper and one lower bound, the agent's true parameter value would be bracketed, and observing further bounds would tighten the bracket. Marking all lower bounds as zeros and all upper bounds as ones would eventually lead to a step function, with the agent's true parameter value located at the step. A real participant, on the other hand, will produce more noisy data: the lower bounds from some decision trials will be above the upper bounds of other decision trials. To make sense of this, we soften the bracketing procedure and estimate the value of $\eta$ where the bounds switch from predominantly lower bounds to predominantly upper bounds. We do this by fitting a logistic curve, which approaches the step function under ideal conditions and whose inflection point serves as our estimate of a real participant's risk aversion parameter.

### Validation and Model checks

**Simulation of synthetic agents.** Prior to data collection, we simulated synthetic agents to assess the accuracy and fidelity of our risk aversion parameter estimation methods. To this end, we simulated responses of synthetic agents with stable (over conditions) risk aversion parameter values $\{(-0.5, -0.5), (0.0, 0.0), (0.5, 0.5), (1.0, 1.0), (1.5, 1.5)\}$, consistent with the EUT model and estimated their parameters separately to check our ability to recover the parameters accurately as well as check for floor or ceiling constraints in our estimation procedure. Also shown are estimates recovered from ground truth risk aversion parameter values consistent with the EE model (*i.e.* $(0, 1)$). For each type of synthetic agent, we simulated 10 copies, all with noise comparable to what was estimated in a previous experiment [5]. All simulations were run for 160 trials, consistent with the setup used on real participants.

**Findings.** In Fig E, we show the estimated parameters for each estimation procedure and data pooling for the different synthetic agents. This shows that we are able to robustly recover parameters across a large space of possible risk aversion parameters.

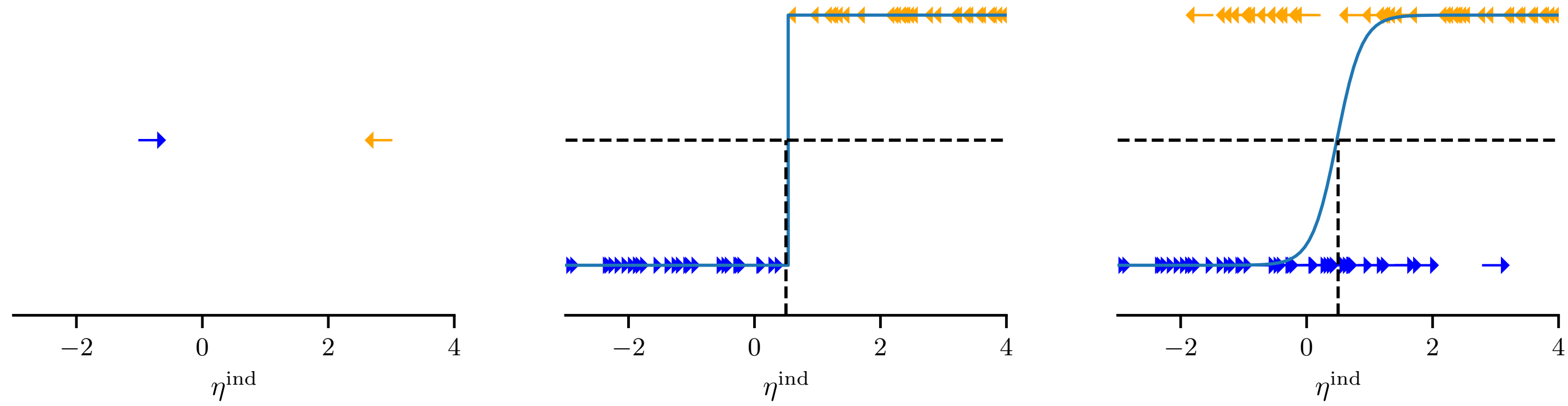


**Fig C. Building the bracketing estimation method. Left,** the choice of a gamble implies either a lower bound (blue arrow) or an upper bound (yellow arrow) on the decision-makers risk aversion parameter at the gamble pair's $\eta^{\mathrm{ind}}$. **Middle,** plotting lower bounds at vertical value 0 and upper bounds at vertical value 1 lead to a step function for an ideal agent, with the agent's true parameter value located at the step. **Right,** To make sense of the noisy data, we soften the bracketing procedure and estimate the value of $\eta$ where the bounds switch from predominantly lower bounds to predominantly upper bounds. We do this by fitting a logistic curve, whose inflection point serves as our point estimate of an agent's risk aversion parameter in the presence of realistic noise. This point estimate is indicated by the vertical dotted black line.

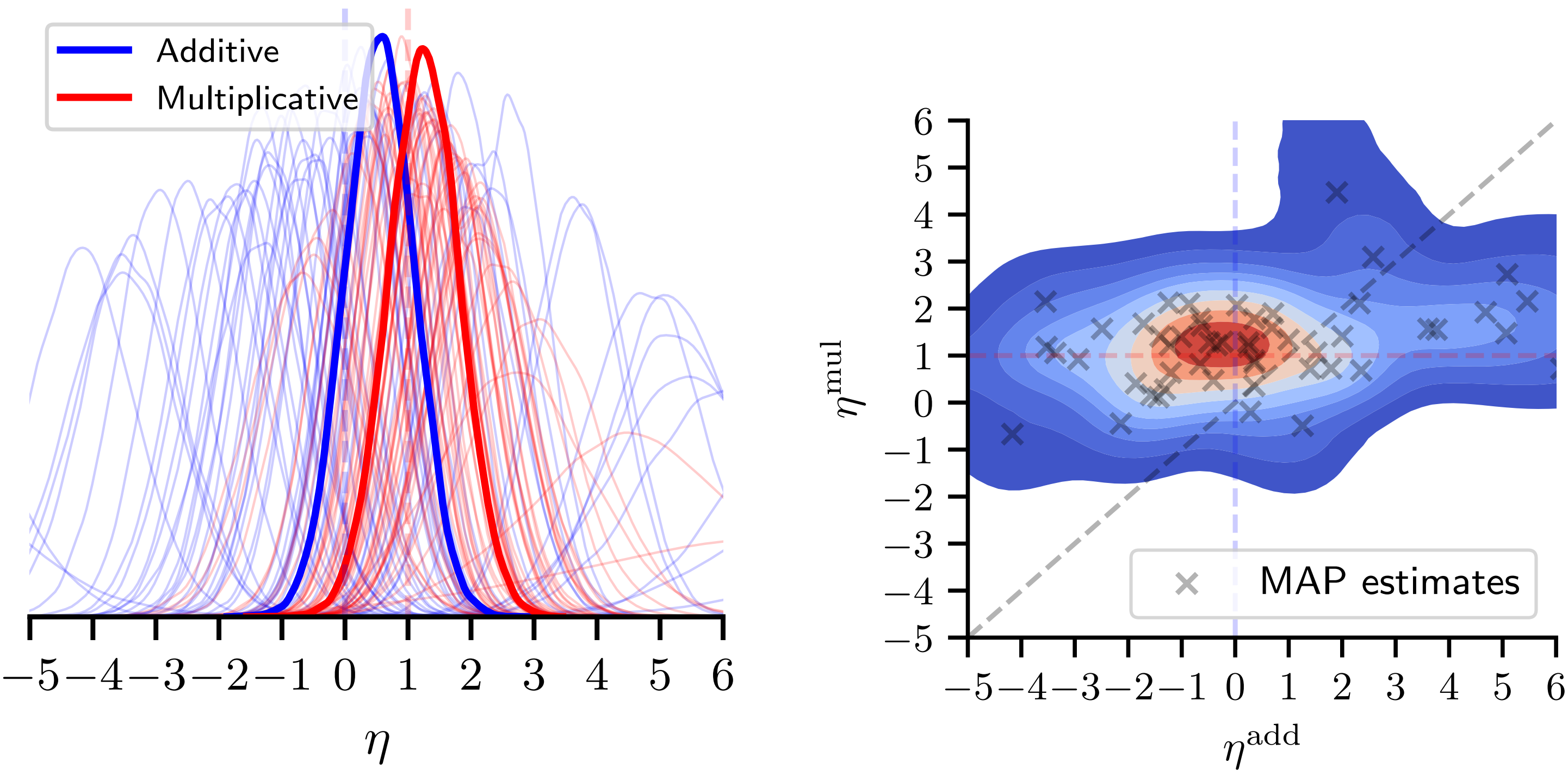


**Fig D. Risk aversion parameter estimates from the bracketing method. a,** the posterior distributions of the risk aversion parameters for additive (blue) and multiplicative (red) conditions. Transparent lines are individual-level estimates, and solid lines are group-level estimates. **b,** the joint distribution of the individual risk aversion parameter estimates in the multiplicative and additive conditions. Blue and red dashed lines indicate the values predicted by the EE model for each condition. Grey dashed line indicates the predictions of the EUT model. X marks the maximum a posteriori (MAP) estimate for each individual's parameter estimation.

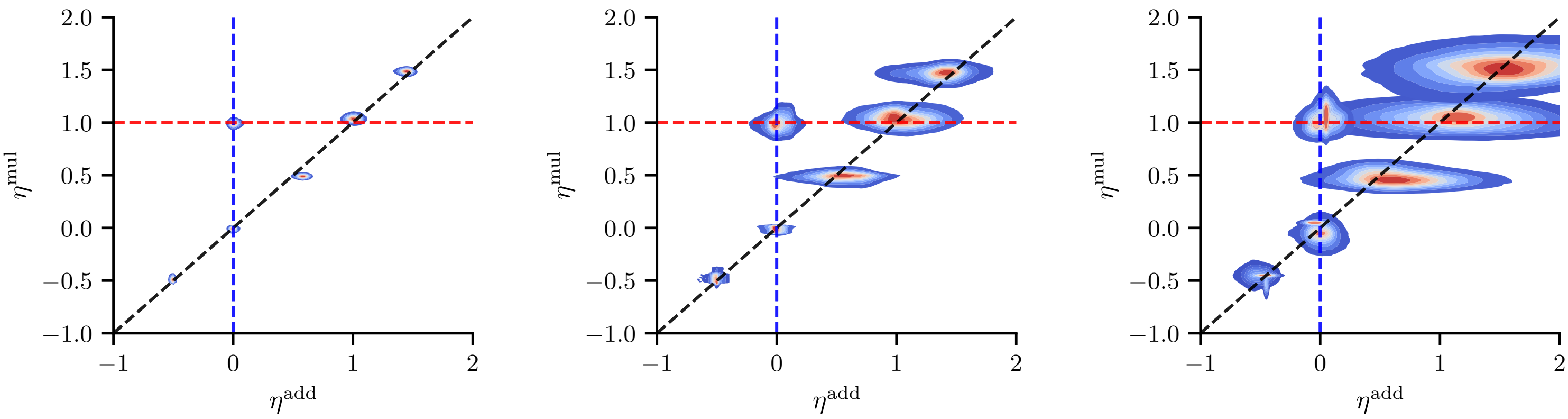


**Fig E. Parameter estimate recovery from synthetic agents.** Individual risk aversion parameter estimates for synthetic agents, recovered via our fitting methods from ground-truth values fixed at $\{(-0.5, -0.5), (0.0, 0.0), (0.5, 0.5), (1.0, 1.0), (1.5, 1.5)\}$, consistent with the EUT model. Also shown are estimates recovered from ground truth values consistent with the EE model (*i.e.*$(0, 1)$). **Left,** Bayesian method partial pooling. **Middle,** Bayesian method no pooling. **Right,** bracketing method no pooling.

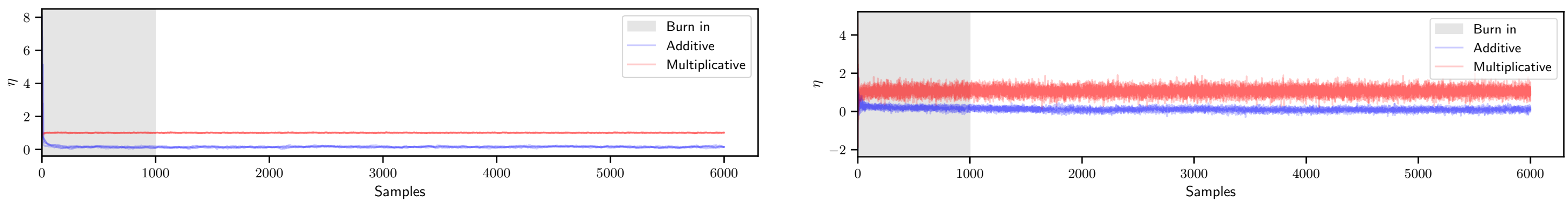


**Fig F. MCMC sampling traces. Left,** traces of risk aversion parameters (additive and multiplicative) for the no pooling model. **Right,** Same but for the partial pooling model.

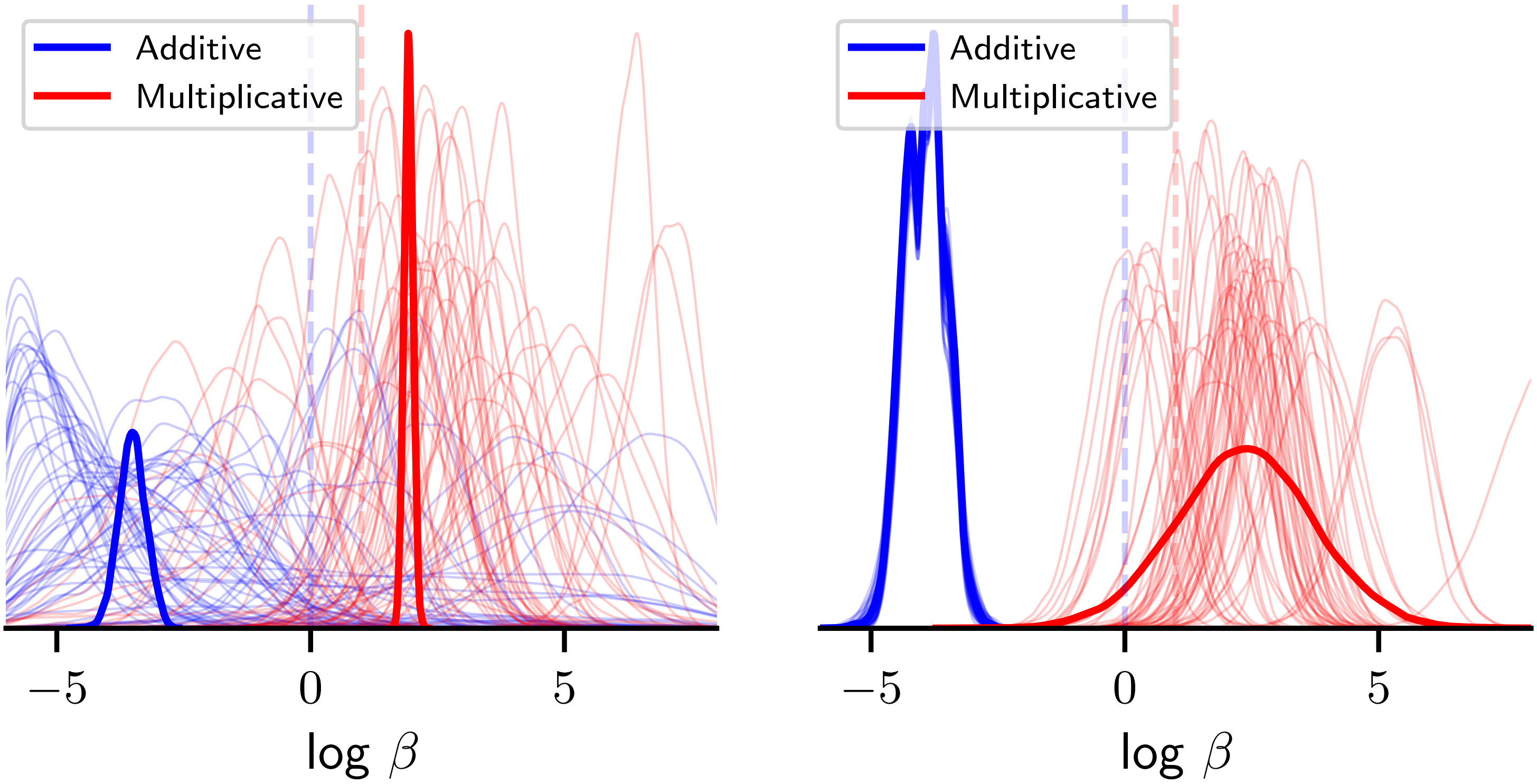


**Fig G. Parameter estimates for the sensitivity parameter. Left,** posterior estimates of the log-sensitivity parameter $\beta$ for the no-pooling model.Transparent lines are individual-level estimates using no pooling, and solid lines are group-level estimates using full pooling. **Right,** same as left but for individual and group estimates of the partial pooling model.

## Appendix D: Results

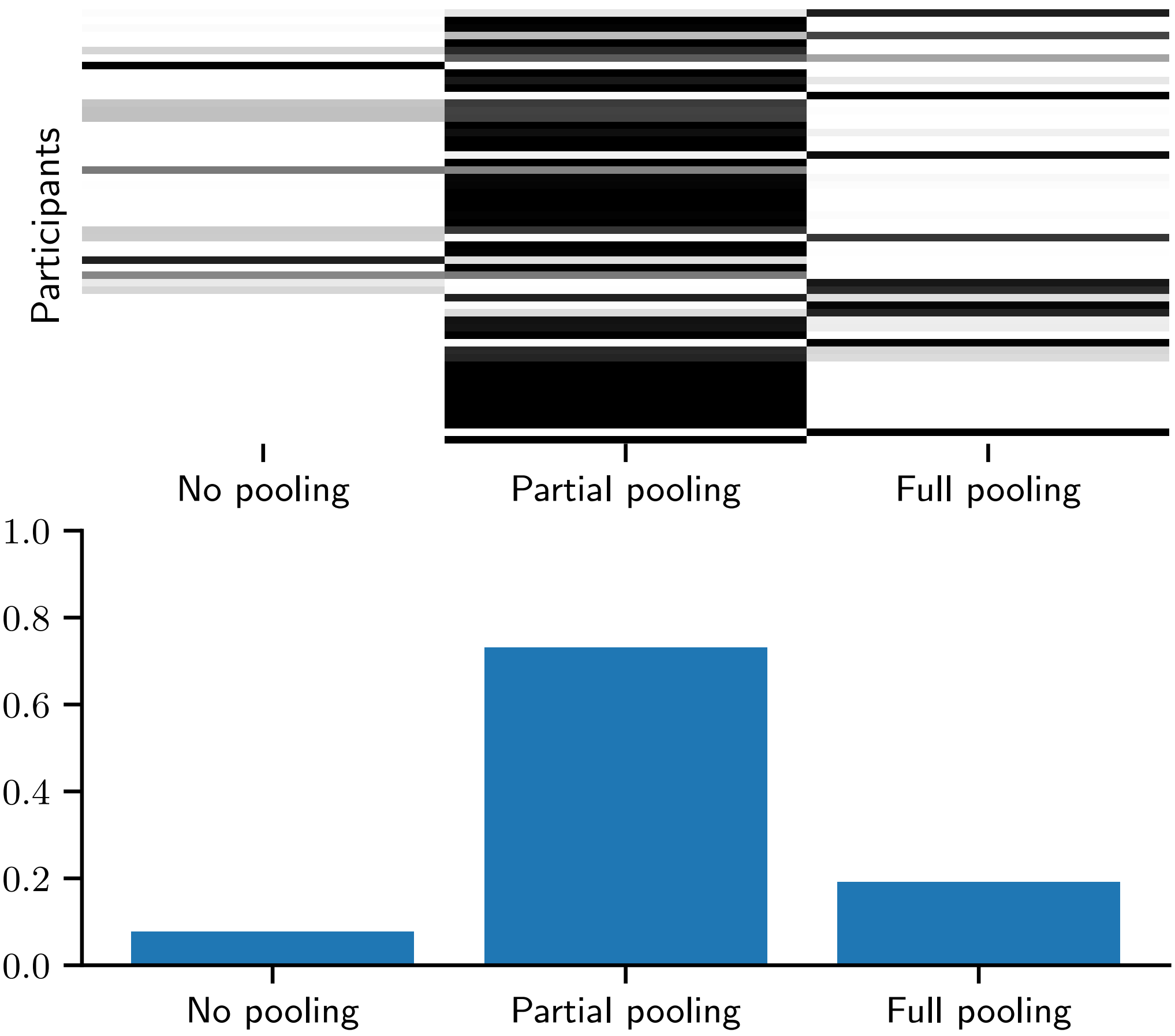


**Fig H. Data pooling comparison. Upper,** posterior model probabilities for each model based on the model indicator variable. **Lower,** The same but summed over all participants.

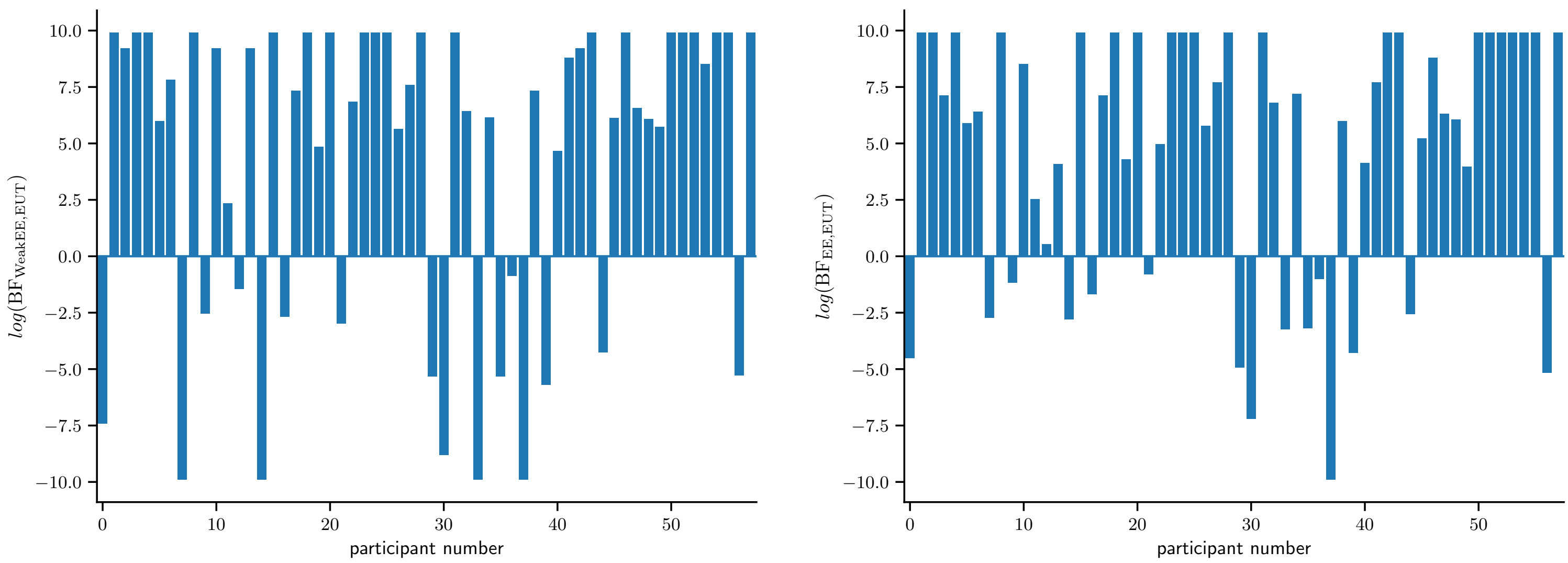


**Fig I. Bayes factor analysis for no pooling data. Left,** Individual log BayesFactors for weak EE vs EUT.**Right,** Individual log BayesFactors for EE vs EUT.

**Confounder analysis.** To assess whether any other differences between the conditions can explain the difference in estimated risk aversion parameters, we used Bayesian linear models. We specified the pairwise difference between the conditions of the risk-aversion parameter as the dependent variable and used the following pairwise differences between conditions as independent variables: 1. Luck, defined as the difference between the average growth rate for the chosen gamble and the realized growth rate, summed over time. 2. Variance, defined as the variance of wealth over time. 3. Log variance, defined as the variance of the log wealth in each condition. 4. Terminal wealth is defined as the wealth at the end of the game. The results show that none of the considered confounding factors have substantive evidence for their inclusion in explaining the observed difference in risk aversion parameters. The variance of log wealth, with $P_{inclusion} = 0.155$, corresponds to a BF $\approx 5.461$ against the inclusion of the factor, meaning there is moderate evidence against its inclusion.

**Bayesian linear regression on risk aversion parameters.** To evaluate the dependency of the condition-specific risk aversion parameters on other variables, including established risk questionnaires, we performed a Bayesian Linear regression. We show the results of this regression in tables B and C, first with the additive condition risk aversion parameter as the target variable. The second set of results is for the equivalent regression with the multiplicative condition risk aversion parameter as the target variable. Third, we also regressed the same set of variables on the change in risk preferences.

**Table A. Regression of potential confounding variables.**

| independent variable | P(B != 0 \|Y) | posterior mean | posterior SD | BF_incl |
|---|---|---|---|---|
| Intercept | 1 | 0.958 | 0.021 | |
| d_luck | 0.052 | 0 | 0 | 0.055 |
| d_final_wealth | 0.072 | 0 | 0 | 0.077 |
| d_log_var_wealth | 0.115 | −0.003 | 0.011 | 0.130 |
| d_var_wealth | 0.051 | 0 | 0 | 0.054 |

**Table B. Regression on additive risk aversion parameter**

| independent variable | P(B != 0 \|Y) | posterior mean | posterior SD | BF_incl |
|---|---|---|---|---|
| Intercept | 1 | 0.102 | 0.006 | |
| X1.0_partial_pooling | 0.895 | 0.111 | 0.054 | 8.508 |
| perception_E | 0.119 | −0.0001 | 0.001 | 0.135 |
| perception_FG | 0.182 | −0.0004 | 0.001 | 0.222 |
| perception_FI | 0.090 | 0 | 0.001 | 0.100 |
| perception_HS | 0.314 | 0.001 | 0.001 | 0.458 |
| perception_R | 0.118 | 0.0001 | 0.001 | 0.134 |
| perception_S | 0.111 | 0.0001 | 0.001 | 0.124 |
| preference_E | 0.197 | −0.0004 | 0.001 | 0.245 |
| preference_FG | 0.085 | 0 | 0.001 | 0.093 |
| preference_FI | 0.241 | 0.001 | 0.002 | 0.318 |
| preference_HS | 0.212 | 0.0005 | 0.001 | 0.269 |
| preference_R | 0.423 | −0.001 | 0.001 | 0.733 |
| preference_S | 0.649 | 0.002 | 0.002 | 1.848 |
| benefits_E | 0.138 | −0.0001 | 0.001 | 0.160 |
| benefits_FG | 0.103 | −0.0001 | 0.001 | 0.115 |
| benefits_FI | 0.587 | 0.002 | 0.002 | 1.421 |
| benefits_HS | 0.182 | 0.0003 | 0.001 | 0.222 |
| benefits_R | 0.441 | −0.001 | 0.001 | 0.789 |
| benefits_S | 0.104 | 0 | 0.0005 | 0.117 |
| risk_propensity | 0.466 | −0.001 | 0.001 | 0.871 |
| Age | 0.101 | 0.0001 | 0.0004 | 0.112 |
| Sex1 | 0.079 | −0.0002 | 0.004 | 0.085 |
| Income | 0.084 | 0 | 0 | 0.092 |

**Table C. Regression on multiplicative risk aversion parameter estimate**

| independent variable | P(B != 0 \|Y) | posterior mean | posterior SD | BF_incl |
|---|---|---|---|---|
| Intercept | 1 | 1.060 | 0.020 | |
| X0.0_partial_pooling | 0.880 | 1.099 | 0.527 | 7.320 |
| perception_E | 0.051 | 0 | 0.001 | 0.054 |
| perception_FG | 0.061 | 0.0002 | 0.002 | 0.065 |
| perception_FI | 0.134 | 0.002 | 0.005 | 0.155 |
| perception_HS | 0.053 | 0.0001 | 0.001 | 0.056 |
| perception_R | 0.050 | 0 | 0.001 | 0.053 |
| perception_S | 0.073 | −0.0003 | 0.002 | 0.079 |
| preference_E | 0.074 | −0.0002 | 0.001 | 0.079 |
| preference_FG | 0.481 | −0.007 | 0.009 | 0.928 |
| preference_FI | 0.445 | −0.006 | 0.007 | 0.803 |
| preference_HS | 0.068 | −0.0002 | 0.001 | 0.073 |
| preference_R | 0.082 | 0.0002 | 0.001 | 0.090 |
| preference_S | 0.054 | −0.0001 | 0.001 | 0.057 |
| benefits_E | 0.058 | −0.0001 | 0.001 | 0.062 |
| benefits_FG | 0.078 | −0.0004 | 0.002 | 0.084 |
| benefits_FI | 0.110 | −0.001 | 0.003 | 0.123 |
| benefits_HS | 0.099 | −0.0005 | 0.002 | 0.110 |
| benefits_R | 0.055 | 0.0001 | 0.001 | 0.058 |
| benefits_S | 0.051 | 0 | 0.001 | 0.053 |
| risk_propensity | 0.082 | −0.0002 | 0.001 | 0.089 |
| Age | 0.126 | −0.001 | 0.002 | 0.144 |
| Sex1 | 0.090 | 0.004 | 0.019 | 0.098 |
| Income | 0.068 | 0 | 0 | 0.073 |

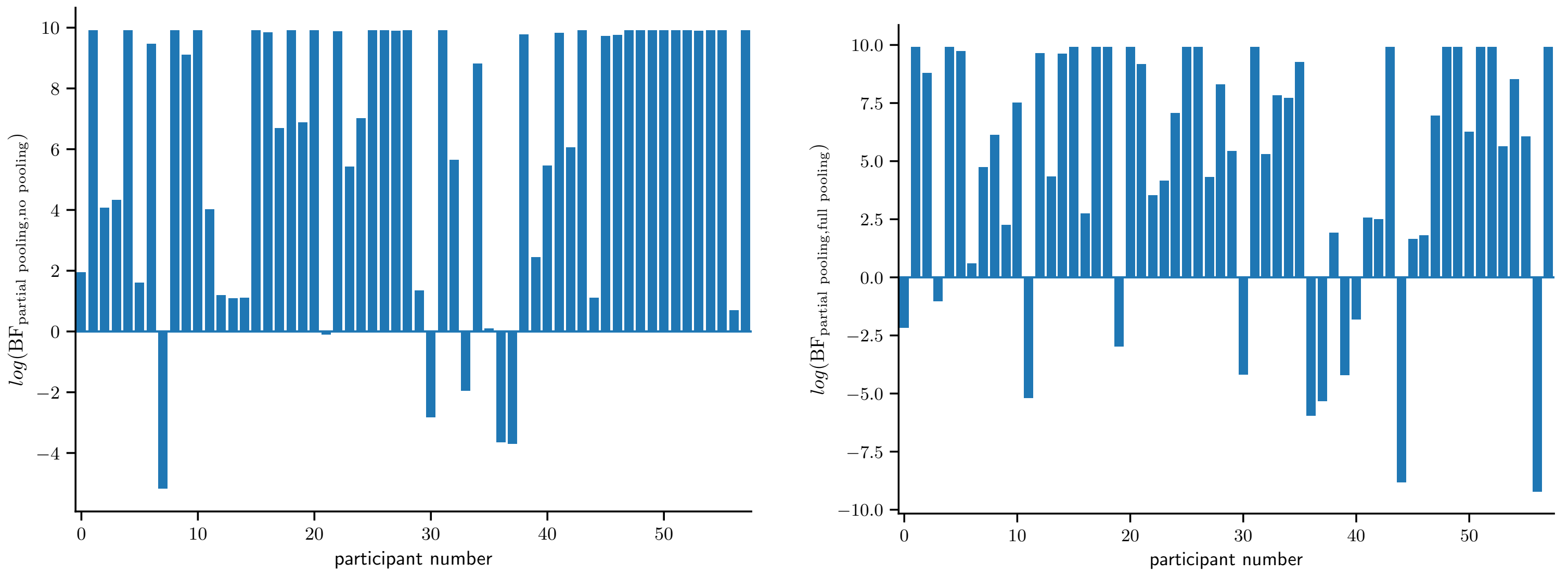


**Fig J. Bayes factor analysis for partial pooling data. Left,** Individual log Bayes Factors for partial pooling vs no pooling.**Right,** Individual log BayesFactors for partial pooling vs full pooling.

**Table D.** regression on delta of risk-preferences

| independent variable | P(B != 0 \|Y) | posterior mean | posterior SD | BF_incl |
|---|---|---|---|---|
| Intercept | 1 | 0.958 | 0.020 | |
| perception_E | 0.026 | 0 | 0.001 | 0.027 |
| perception_FG | 0.031 | 0.0001 | 0.001 | 0.032 |
| perception_FI | 0.086 | 0.001 | 0.004 | 0.095 |
| perception_HS | 0.028 | 0 | 0.001 | 0.029 |
| perception_R | 0.025 | 0 | 0.001 | 0.026 |
| perception_S | 0.040 | -0.0002 | 0.001 | 0.041 |
| preference_E | 0.043 | -0.0002 | 0.001 | 0.044 |
| preference_FG | 0.388 | -0.006 | 0.009 | 0.634 |
| preference_FI | 0.298 | -0.004 | 0.007 | 0.424 |
| preference_HS | 0.038 | -0.0001 | 0.001 | 0.040 |
| preference_R | 0.032 | 0.0001 | 0.001 | 0.033 |
| preference_S | 0.025 | 0 | 0.001 | 0.026 |
| benefits_E | 0.031 | -0.0001 | 0.001 | 0.032 |
| benefits_FG | 0.042 | -0.0002 | 0.001 | 0.044 |
| benefits_FI | 0.047 | -0.0003 | 0.002 | 0.049 |
| benefits_HS | 0.050 | -0.0003 | 0.002 | 0.052 |
| benefits_R | 0.026 | 0 | 0.0005 | 0.027 |
| benefits_S | 0.026 | 0 | 0.0005 | 0.026 |
| risk_propensity | 0.048 | -0.0002 | 0.001 | 0.051 |
| Age | 0.070 | -0.0003 | 0.001 | 0.075 |
| Sex1 | 0.053 | 0.003 | 0.016 | 0.056 |
| Income | 0.037 | 0 | 0 | 0.038 |

**Reinforcement heuristic.** Because outcomes (and current wealth) are shown on every trial, it is possible in principle that simple trial-to-trial reinforcement heuristics based on the previous trial's outcome could influence subsequent choices. Because gambles were randomly assigned to left/right positions on the screen, side-based staying is uninformative about image- or gamble-level reinforcement. We therefore also analysed reinforcement heuristics at the level of features of the chosen gamble. Staying behaviour was tested along three features of the previous trial: (i) side, (ii) gamble variance, and (iii) gamble variance conditional on having chosen the higher-variance gamble on the previous trial. For each feature and each condition we computed the per-participant paired difference between rewarded and non-rewarded previous trials on logit-transformed choice proportions, tested it with a paired t-test using pingouin[**?** ] (v0.5.5), and report Bayes factor ($\mathrm{BF}_{01}$, Cauchy scale-factor $r = 0.707$) against the null of no reward effect (six tests total; 3 features $\times$ 2 conditions). Paired differences are centred close to zero and Bayes factors favour the null with moderate evidence in all but one case ((i) additive seesion, $BF_{01} = 6.49$, $t(57) = -0.379$, $95\%\ CI = [-0.13, 0.09]$, multiplicative session, $BF_{01} = 6.94$, $t(57) = 0.109$, $95\%\ CI = [-0.11, 0.1]$; (ii) additive seesion, $BF_{01} = 5.75$, $t(57) = -0.637$, $95\%\ CI = [-0.15, 0.08]$, multiplicative session, $BF_{01} = 6.76$, $t(57) = -0.257$, $95\%\ CI = [-0.13, 0.1]$; (iii) additive seesion, $BF_{01} = 6.90$, $t = -0.158$, $95\%\ CI = [-2.67, 2.28]$) in the conditional-variance case of the multiplicative session there is not enough evidence for or against the null ((iii), multiplicative session, $BF_{01} = 0.56$, $t(57) = -2.348$, $95\%\ CI = [-0.37, -0.03]$) to make a strong conclusion (Fig K). Taken together, these analyses suggest that trial-wise reinforcement did not induce a simple reinforcement heuristic at the level of side, variance, or conditional variance that could account for the main result.

**Learning reliability checks.** A potential concern is that, because each stimulus image is encountered only 15 times during the learning phase, participants may form a less reliable (potentially more uncertain) mapping in the multiplicative condition than in the additive condition. This would be putatively because the increment size is the same in the additive condition but wealth dependent in the multiplicative. To test this

| | Hypothesis | T | dof | alternative | p-val | CI95% | cohen-d | BF10 | power | BF01 |
|---|---|---|---|---|---|---|---|---|---|---|
| T-test | P(stay side \| eta = 0, previous win = 1) != P(... | -0.378914 | 57 | two-sided | 0.706160 | [-0.13, 0.09] | 0.054627 | 0.154 | 0.069380 | 6.493506 |
| T-test | P(stay \| eta = 1, previous win = 1) != P(stay ... | -0.108961 | 57 | two-sided | 0.913616 | [-0.11, 0.1] | 0.014683 | 0.144 | 0.051386 | 6.944444 |
| T-test | P(stay variance \| eta = 0, previous win = 1) !... | -0.636565 | 57 | two-sided | 0.526957 | [-0.15, 0.08] | 0.086840 | 0.174 | 0.099674 | 5.747126 |
| T-test | P(stay variance\| eta = 1, previous win = 1) !=... | -0.257040 | 57 | two-sided | 0.798074 | [-0.13, 0.1] | 0.036579 | 0.148 | 0.058639 | 6.756757 |
| T-test | P(stay risky \| eta = 0, previous win = 1) != P... | -0.157649 | 57 | two-sided | 0.875290 | [-2.67, 2.28] | 0.029082 | 0.145 | 0.055450 | 6.896552 |
| T-test | P(stay risky \| eta = 1, previous win = 1) != P... | -2.348483 | 57 | two-sided | 0.022342 | [-0.37, -0.03] | 0.312435 | 1.789 | 0.647752 | 0.558971 |

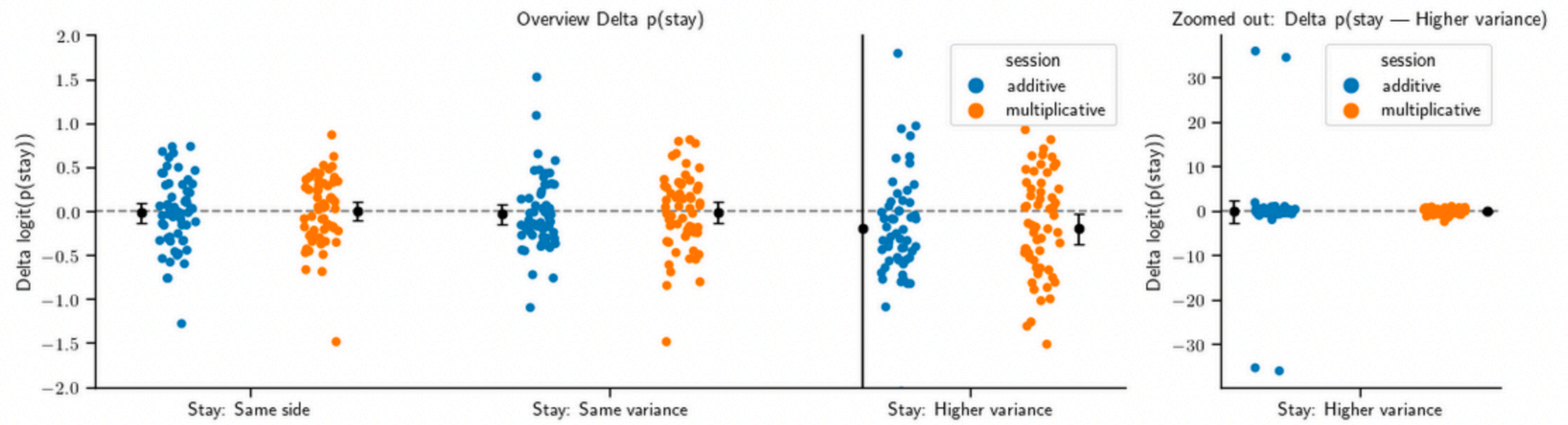


**Fig K. Paired differences in stay probabilities by previous-trial reinforcement.** Strip-plots of paired differences in the logit-transformed stay probabilities between previously rewarded and non-rewarded trials, with paired t-test statistics (mean and 95% confidence intervals). Left panel: the three hypotheses tested per session. Left: does reward on the previous trial affect whether participants choose the same side on the current trial. Middle: does reward on the previous trial affect whether participants choose the same gamble variance on the current trial. Right: does reward on the previous trial affect whether participants continue choosing the higher-variance gamble, given they selected a high-variance gamble on the previous trial. Right panel: zoomed-out version of the high-variance plot, as some values fall outside the axis limits of the left panel.

directly, we quantified learning reliability using (i) accuracy on "no-brainer" trials. For each participant and condition, we computed the proportion of no-brainer trials answered correctly and the differences between the conditions in this accuracy (Fig L). As can be seen the difference in accuracy centers on zero indicating no systematic difference between the two conditions. Formally this is tested as Bayesian one-sampled t-tests against 0 (for the run average performance, $BF_{01} = 7.3,\ t(65) = 0.19,\ 95\%\ CI = [-0.03, 0.03]$). For this analysis we also included the eight participants that were excluded from the main analysis due to poor performance.

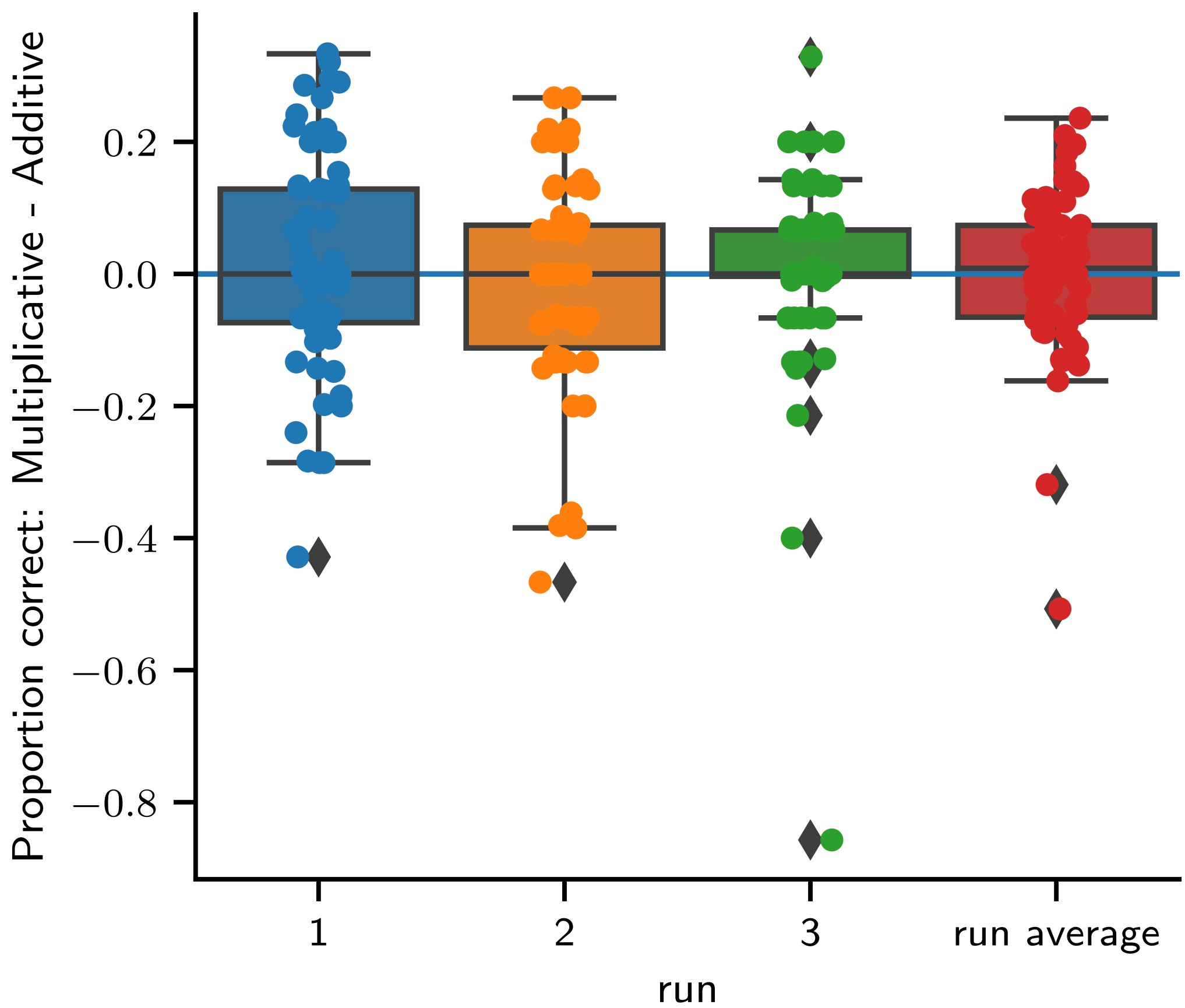


**Fig L. Difference in no-brainer accuracy (proportion correct, additive vs. multiplicative), shown by run and averaged across runs.** Plots show 5 number summaries for the group with dots indicating each subject's performance.

**Learning effects during the decision task.** To assess whether learning proceeded during the decision task, and if this impacted on the change in risk preference we estimated the risk aversion parameters based only on 1st half of the decision phase versus 2nd half. We find no substantive

differences between the two, as would be expected if there was still substantive learning ongoing. The same shift in risk preference is present, and in both cases it aligns quantitatively as an approximation to the time optimal solution. This is evident in Fig M, where the left panel shows the estimation of risk preferences based on the first half and the right panel shows the equivalent from the second.

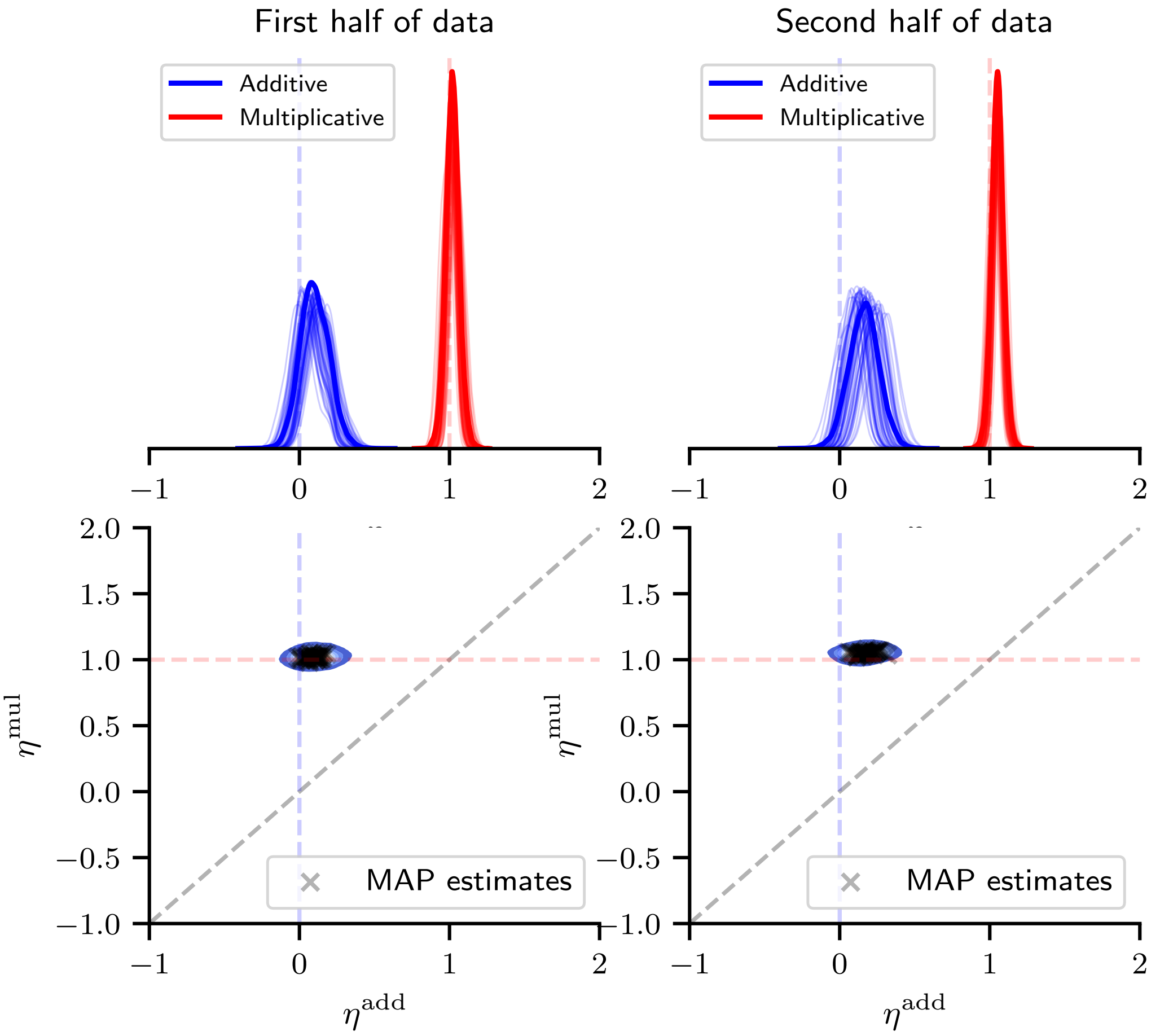


**Fig M. Bivariate distribution of posterior risk preference parameter for both additive and multiplicative conditions.** Left, 1st half of decision trials, right, 2nd half of trials. Fiducials indicate theoretical predictions with blue indicating time optimal strategy for the additive condition, red the equivalent strategy for the multiplicative conditions, and the diagonal gray the predicted strategy under a dynamic invariant EUT model.

## References


1. Skjold, B., Steinkamp, S. R., Connaughton, C., Hulme, O. J. & Peters, O. Are risk preferences optimal? (registered report), DOI: 10.31219/osf.io/ew2sx (2023).

2. Markowitz, H. The utility of wealth. J. Polit. Econ. **60**, 151–158, DOI: 10.1086/257177 (1952).

3. Stefan, A. M., Gronau, Q. F., Schönbrodt, F. D. & Wagenmakers, E.-J. A tutorial on bayes factor design analysis using an informed prior. Behav. Res. Methods **51**, 1042–1058, DOI: 10.3758/s13428-018-01189-8 (2019).

4. Stefan, A. M. & Schönbrodt, F. D. Bfda: An r package for bayes factor design analysis. GitHub (2018).

5. Meder, D. et al. Ergodicity-breaking reveals time optimal decision making in humans. PLOS Comput. Biol. **17**, 1–25, DOI: 10.1371/journal.pcbi.1009217 (2021).

6. Ovalle-Fresa, R., Di Pietro, S. V., Reber, T. P., Balbi, E. & Rothen, N. Standardized database of 400 complex abstract fractals. Behav. Res. Methods **54**, 2302–2317, DOI: 10.3758/s13428-021-01726-y (2022).

7. Price, J. & Connaughton, C. Distinguishing risk preferences using repeated gambles, DOI: 10.48550/arXiv.2308.07054 (2023).

8. Coller, M. & Williams, M. B. Eliciting individual discount rates. Exp. Econ. **2**, 107–127, DOI: 10.1023/A:1009986005690 (1999).

9. Harrison, G. W. & Elisabet Rutström, E. Risk Aversion in the Laboratory, vol. 12 of Research in Experimental Economics, 41–196 (Emerald Group Publishing Limited, 2008).
