# Supplementary material for: Ergodicity transformations predict human decision-making under risk
Source: PLoS Comput Biol. 2026 Jul 20;22(7):e1014409. doi: 10.1371/journal.pcbi.1014409 (PMC13472505; doi:10.1371/journal.pcbi.1014409)
Supplement: Supplementary file 2 [file pcbi.1014409.s002.pdf]

Editor letter.

PCOMPBIOL-D-25-01780
Ergodicity transformations predict human decision-making under risk
PLOS Computational Biology

Dear Dr. Hulme,

Thank you for submitting your manuscript to PLOS Computational Biology. After careful consideration, we feel that it has merit but does not fully meet PLOS Computational Biology's publication criteria as it currently stands. Therefore, we invite you to submit a revised version of the manuscript that addresses the points raised during the review process.

Please submit your revised manuscript by Jan 19 2026 11:59PM. If you will need more time than this to complete your revisions, please reply to this message or contact the journal office at ploscompbiol@plos.org. When you're ready to submit your revision, log on to https://www.editorialmanager.com/pcompbiol/ and select the 'Submissions Needing Revision' folder to locate your manuscript file.

Please include the following items when submitting your revised manuscript:

* A rebuttal letter that responds to each point raised by the editor and reviewer(s). You should upload this letter as a separate file labeled **'Response to Reviewers**'. This file does not need to include responses to formatting updates and technical items listed in the 'Journal Requirements' section below.
* A marked-up copy of your manuscript that highlights changes made to the original version. You should upload this as a separate file labeled **'Revised Manuscript with Track Changes'.**
* An unmarked version of your revised paper without tracked changes. You should upload this as a separate file labeled **'Manuscript'.**

If you would like to make changes to your financial disclosure, competing interests statement, or data availability statement, please make these updates within the submission form at the time of resubmission. Guidelines for resubmitting your figure files are available below the reviewer comments at the end of this letter

We look forward to receiving your revised manuscript.

Kind regards,

Jian Liu
Academic Editor
PLOS Computational Biology

Natalia Komarova
Section Editor
PLOS Computational Biology

Additional Editor Comments:

The manuscript requires significant revisions to strengthen its contribution. The **relationship to previous work** needs clarification to better **highlight the novel contribution of this study.** The **rationale for the chosen design** requires a more detailed justification. **Additional analysis** would help to fully support the conclusions drawn.

Reply to the editor.

We have now clarified how this work relates to and extends, Meder et al., and revised the Introduction and Discussion to make that relationship explicit. There are three advantages to this new work: 1) replication, 2) more careful controls on how the task is learnt, 3) making the gambles more realistic by making them consequential.

1. Replication. In the revised manuscript (in Introduction and Discussion) we now explain how the work provides a high-powered replication of Meder et al., increasing the power with more than threefold the sample size from an n=18 to n=58, with a total of 168 experimental trials per session and 336 per subject. We argue that replications of this sort are critical in behavioral science, where there are substantive concerns about the replicability of findings. We therefore aimed to provide a rigorous, higher-powered test of the original effect under an improved paradigm.

Furthermore, this study was fully preregistered, with all primary analyses written ahead of time. The replication is twofold, in the sense that we report two datasets, one pilot and one larger dataset, both of which provide consistent patterns of model evidence in line with the original finding.

2. Learning session. In this new study, we address criticisms that targeted the learning session, whereby wealth trajectories were bounded and controlled differently between the two conditions. Here we make sure that this is not the case. In the learning task, images are sampled randomly with equal probability, and trajectories are not artificially shaped or bounded differently across conditions. In other words, the learning experience is matched across dynamics by design.

3. Gamble realisation. Finally, the original study was criticised for not realising all trials, which made it ambiguous what the current wealth was. For this reason, we converted the study so that every trial was realised in real time so current wealth is always well-defined, salient, and available to participants at the moment of choice.

In summary. Our prior work in Meder et al. was a proof-of-concept study that challenged a central tenet of decision science: the stability of risk preferences. In this study, we have performed a higher powered, preregistered experiment that explicitly addresses each of the key methodological criticisms that were levelled at the original study. The current study thus provides a rigorous replication and generalization of the original finding.

Taken together, these revisions sharpen the interpretation: the observed shift in fitted utility functions is not attributable to differential learning or ambiguity about wealth, but tracks the experimentally manipulated reward dynamics in accordance with the maximization of time average growth rates.

Journal Requirements:

1) We ask that a manuscript source file is provided at Revision. Please upload your manuscript file as a .doc, .docx, .rtf or .tex. If you are providing a .tex file, please upload it under the item type u2018LaTeX Source Fileu2019 and leave your .pdf version as the item type u2018Manuscriptu2019.

2) Please provide an Author Summary. This should appear in your manuscript between the Abstract (if applicable) and the Introduction, and should be 150-200 words long. The aim should be to make your findings accessible to a wide audience that includes both scientists and non-scientists. Sample summaries can be found on our website under Submission Guidelines:
https://journals.plos.org/ploscompbiol/s/submission-guidelines#loc-parts-of-a-submission

Why do people sometimes take risks and other times play it safe? Classic decision theories often assume each person has a stable “utility function”—a fixed way of valuing gains and losses—and that people choose options that maximize expected utility. Recent theory suggests this picture can fail when the real goal is to grow resources over time. In particular, the strategy that maximizes long-run growth can depend on how wealth changes: sometimes gains and losses add up (additive dynamics), while in other settings outcomes compound (multiplicative dynamics). Growth-optimization theory predicts that the utility function that best describes behavior should therefore shift with the underlying wealth dynamics. We tested this idea in a fully consequential risky decision-making experiment in which participants experienced different wealth dynamics and their wealth was updated on every trial. Using Bayesian models, we estimated participants’ utility functions separately under each dynamic. We found strong evidence that people change their risk taking under these different conditions. These results suggest that human risk-taking is not governed by a single fixed preference curve, but can adapt rapidly to the dynamical context in a way consistent with long-run growth optimization.

3) Please upload all main figures as separate Figure files in .tif or .eps format. For more information about how to convert and format your figure files please see our guidelines:
https://journals.plos.org/ploscompbiol/s/figures

4) We notice that your supplementary Figures, Tables, and information are included in the manuscript file. Please remove them and upload them with the file type 'Supporting Information'. Please ensure that each Supporting Information file has a legend listed in the manuscript after the references list.

5) Please amend your detailed Financial Disclosure statement. This is published with the article. It must therefore be completed in full sentences and contain the exact wording you wish to be published.
- State the initials, alongside each funding source, of each author to receive each grant. For example: "This work was supported by the National Institutes of Health (####### to AM; ###### to CJ) and the National Science Foundation (###### to AM)."

Done.

- State what role the funders took in the study. If the funders had no role in your study, please state: "The funders had no role in study design, data collection and analysis, decision to publish, or preparation of the manuscript.".
If you did not receive any funding for this study, please simply state: u201cThe authors received no specific funding for this work.u201d

Done.

Reviewers' comments:

Comments to the Authors:

Please note here if the review is uploaded as an attachment.

Reviewer #1: The paper by Skjold et al. aimed to provide a mechanistic explanation for the context specific risk preferences by participants. Namely, during the additive and multiplicative environments, subjects don't have stable risk preference, instead, their risk attitudes are driven by accruing wealth in the fastest manner through ergodicity transformation. They provided model comparison results between the standard utility (EUT) model and the ergodicity transformation (EE) model. My concerns regarding this manuscripts are listed below:

1. As the author correctly pointed out, the comparison between the additive and multiplicative decision environments have been tested before and similar results were obtained (Meder et al., 2021), so I'm not fully convinced whether the current manuscript provided enough novelty to the extant literature.

We agree that novelty is an important criterion in scientific publishing. We have revised the manuscript to clarify how the present study advances Meder et al. (2021). While the qualitative prediction has therefore been tested previously, this work provides a substantially stronger and methodologically cleaner test: (i) a higher-powered replication (n=58 vs n=18) that was fully preregistered, and (ii) a principled generalization that directly addresses key methodological criticisms of the original design (matched learning across dynamics and fully consequential trial-by-trial wealth updates). In this sense, the present manuscript is not intended as a novelty-only contribution, but as a rigorous replication and quantitative sharpening of a theoretically constrained account. We would also argue that replication is important in the current context of behavioral and psychological science. Large-scale replication efforts indicate that many published effects do not reproduce at their originally reported strength (e.g., Open Science Collaboration, 2015; Camerer et al., 2016, 2018). Against this backdrop, we argue that careful replication and quantitative sharpening of theoretically surprising results is a meaningful contribution in its own right—especially when they bear on foundational assumptions such as the stability of utility. Accordingly, we now motivate the manuscript explicitly as a preregistered, higher-powered replication and generalization of Meder et al., designed to remove confounds in learning and ambiguity about trial-wise wealth while testing the same core theoretical prediction.

The paper is now changed to make this argument more clear. The introduction now contains the following paragraph:

Because the qualitative prediction has been tested previously, the contribution of the present study is not a new effect, but a stronger and more diagnostic test of the same core mechanism under cleaner experimental control. Specifically, we treat this work as a preregistered, higher-powered conceptual replication and principled generalization of \cite{MederETAL2021}, designed to reduce ambiguity about what participants experience and to tighten the mapping between the theoretical intervention (wealth dynamics) and observable choice. To this end, the present design addresses methodological concerns raised about earlier implementations, including potential differences between conditions in anticipated wealth, ambiguity about trial-wise wealth trajectories, and the degree to which choices are consequential. Here, wealth is displayed and updated directly during learning and decision-making, and decisions in the main task have immediate trial-by-trial consequences for in-game wealth, with payoffs proportional to final in-game wealth. In addition, learning and decision demands are matched as closely as possible across dynamics, and identifiability of parameters and models was validated via extensive simulation-based recovery (Appendix C). Together, these changes sharpen quantitative model adjudication between the EUT and EE accounts relative to prior work. More broadly, given ongoing concerns about reproducibility in behavioural and psychological science, we view preregistered replication and quantitative sharpening of theoretically constrained, counterintuitive predictions as an important contribution—especially when they bear on foundational assumptions such as the stability of utility.

2. In the discussion, the authors pointed out that one of the drawbacks of the Meder et al. (2021) paper was that "wealth and gamble outcomes were hidden from the participants" and to avoid that, the current manuscript provided participants with the accumulated wealth for each trial. However, by providing feedbacks, the decision trials are not solely driven by participants risk attitudes (either context dependent or independent) anymore. Feedbacks after each trials would act as strong incentive to influence their subsequent trials. One way to test this is to look at subjects' choice preference after winning or losing in the previous trial. To me, not providing feedback in the original paper was a well thought-out move rather than a drawback.

Thank you, this is an important point. The relevant section of the Supplementary results section (Appendix D) now reads:

Reinforcement heuristic. Because outcomes (and current wealth) are shown on every trial, it is possible in principle that simple trial-to-trial reinforcement heuristics (e.g., win–stay / lose–shift) could influence subsequent choices. We therefore tested directly whether participants showed systematic win–stay and/or lose–shift behavior at the level of the chosen gamble (left vs. right). We thus computed the following:

a) Win-stay probability: P (stay | previous win): where stay means choosing the same side as on previous trial
b) Lose-shift probability P (shift | previous loss): where shift means choosing the opposite side to the previous trial

Under win-stay and lose-shift, these probabilities should exceed 0.5. For completion, the converse heuristic (lose-stay, win-shift) predicts these probabilities should be less than 0.5. Across participants, both quantities were centered close to 0.5, with no consistent bias in either direction (Fig. S11). We quantified this using a Bayesian t-test (group-level test against 0.5), which provided evidence in favor of the null hypothesis of no systematic win–stay (η=0: BF_{01}=5.43, 95% CI = [0.48, 0.53]; η=1: BF_{01}=3.12, 95% CI = [0.49, 0.53]) or lose–shift tendency (η=0: BF_{01}=3.47, 95% CI = [0.47, 0.51]; η=1: BF_{01}=4.74,95% CI = [0.46, 0.51]). Taken together, these analyses suggest that trial-wise reinforcement did not induce a simple reinforcement heuristic that could account for the main result.

	Hypothesis	T	dof	alternative	p-val	CI95%	cohen-d	BF10	power	BF01
**T-test**	P(stay | eta = 0, previous win = 0) != 0.5	1.214370	57	two-sided	0.229613	[0.49, 0.53]	0.159455	0.288	0.222645	3.472222
**T-test**	P(stay | eta = 1, previous win = 0) != 0.5	0.902299	57	two-sided	0.370695	[0.49, 0.54]	0.118478	0.211	0.143886	4.739336
**T-test**	P(stay | eta = 0, previous win = 1) != 0.5	0.723429	57	two-sided	0.472375	[0.48, 0.53]	0.094991	0.184	0.109673	5.434783
**T-test**	P(stay | eta = 1, previous win = 1) != 0.5	1.307233	57	two-sided	0.196383	[0.49, 0.53]	0.171648	0.321	0.250518	3.115265

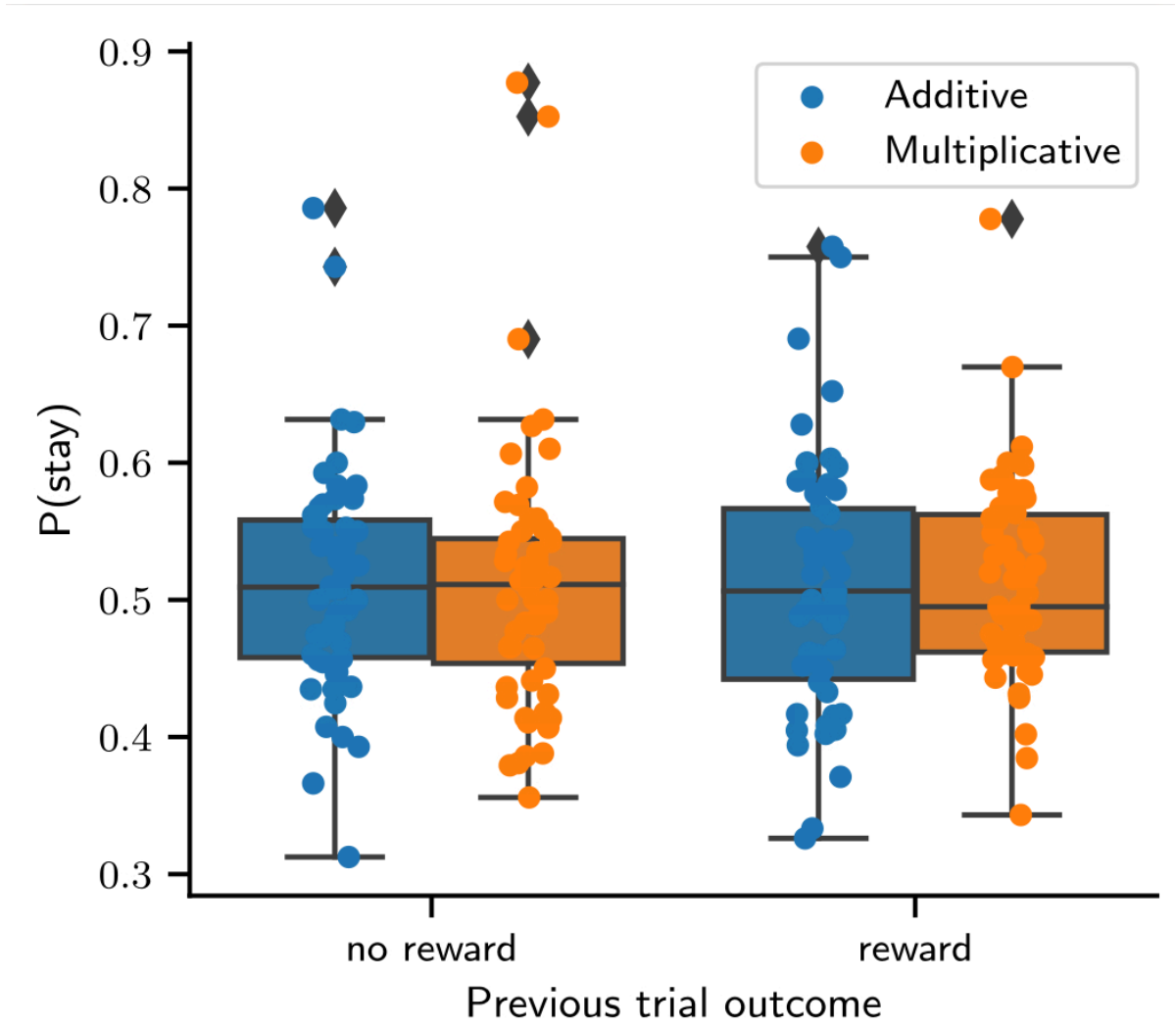

Figure S11 | Button press biases as a function of previous reinforcement. Graph shows choice proportion for staying as a function of that response being rewarded or not in the last trial, for the additive and multiplicative conditions. Five number summary for the group, with dots indicating choice frequencies for each subject.

3. Have the author tested how reliable/accurate about the outcomes from the learning phases? Given there were only 5 repetitions for each image and subjects' wealth level in each trial is likely to be different, participants might turn out to have a more vague estimation of the multiplicative factor than that of the the additive amount for the images. Previous research in cognitive neuroscience have shown how the belief of the environment uncertainty might have an impact on the speed of learning (e.g. McGuire et al., 2014 Neuron) and this might also affect the difference in the later comparison between the additive and multiplicative comparisons.

Thank you. Just to clarify there were ~15 repetitions not 5, however we agree with the spirit of the question. We have now changed the paper to address these points, in the supplementary analysis (Appendix D):

Learning reliability checks. A potential concern is that, because each stimulus image is encountered only 15 times during the learning phase, participants may form a less reliable (potentially more uncertain) mapping in the multiplicative condition than in the additive condition. This would be putatively because the increment size is the same in the additive condition but wealth dependent in the multiplicative. To test this directly, we quantified learning reliability using (i) accuracy on “no-brainer” trials. For each participant and condition, we computed the proportion of no-brainer trials answered correctly and the differences between the conditions in this accuracy (Fig. S.12). As can be seen the difference in accuracy centers on zero indicating no systematic difference between the two conditions. Formally this is tested as Bayesian one-sampled t-tests against 0 (for the run average performance, BF_{01}=7.3, t=0.19, 95% CI [-0.03,0.03]). For this analysis we also included the eight participants that were excluded from the main analysis due to poor performance.

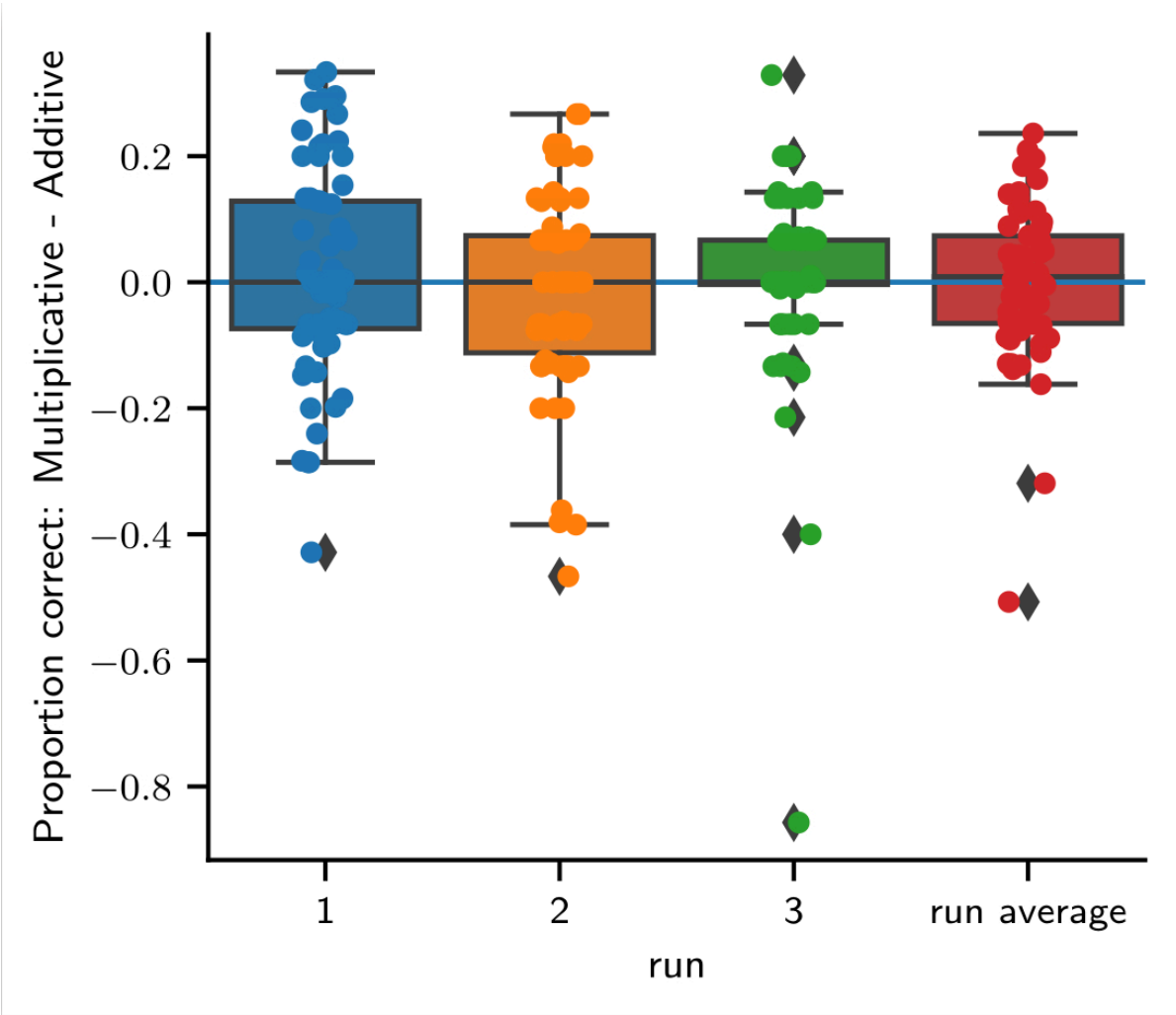

	Run	T	dof	alternative	p-val	CI95%	cohen-d	BF10	power	BF01
**T-test**	Average	0.191632	65	two-sided	0.848628	[-0.03, 0.03]	0.023588	0.137	0.054094	7.29927

Fig. S.12 | Difference in no-brainer accuracy (proportion correct, additive vs. multiplicative), shown by run and averaged across runs. Plots show 5 number summaries for the group with dots indicating each subject's performance.

We also added this summary of results to the discussion:

"Learning-phase uncertainty as an alternative explanation. A potential alternative account is that participants learned the multiplicative mapping less reliably (i.e., with greater uncertainty) due to greater uncertainty because the increments are wealth dependent, and that this differential uncertainty influenced subsequent learning of the fractals. We find no support for this explanation: no-brainer accuracy was comparable across additive and multiplicative conditions (Fig. X). Thus, the observed decision-phase differences are unlikely to be an artifact of poorer or noisier learning in one condition."

4. The authors did perform exploratory analysis to control for the effects of potential confounders such as variance in wealth, or log wealth. However, since the range of wealth (or monetary prospects) in the decision tasks are dramatically different between the additive and multiplicative contexts and it's well established that the utility is marginally decreasing, I'm concerned whether the results and estimated parameters are reliable enough if candidate utility models are also included and compared against. For example, what about constructing a EUT model that takes into account the law of diminishing marginal return (LDMR)

The isoelastic utility model is a general model that accommodates marginally decreasing utility, marginally increasing utility, and everything in-between. The law of diminishing marginal return is therefore already included in our EUT candidate model via the free curvature parameter that controls how marginal utility changes with wealth. Our model comparison indicates that this EUT (isoelastic) model is predictively inferior to the EE model.

or a model that considers the contribution of learning components in the pre-assumed "decision" task?

Thanks. We agree the effects of learning are worth exploring because during the decision task subjects continue to experience fractals effects on wealth and thus they can carry on learning. To address the possibility of continued learning we estimate risk aversion parameters for the 1st half versus the 2nd half of the decision trials. We now have a section in the Supplemental analysis (Appendix D) section as follows:

Learning effects during the decision task. To assess whether learning proceeded during the decision task, and if this impacted on the change in risk preference we estimated the risk aversion parameters based only on 1st half of the decision phase versus 2nd half. We find no substantive differences between the two, as would be expected if there was still substantive learning ongoing. The same shift in risk preference is present, and in both cases it aligns quantitatively as an approximation to the time optimal solution. This is evident in Fig. S.13, where the left panel shows the estimation of risk preferences based on the first half and the right panel shows the equivalent from the second.

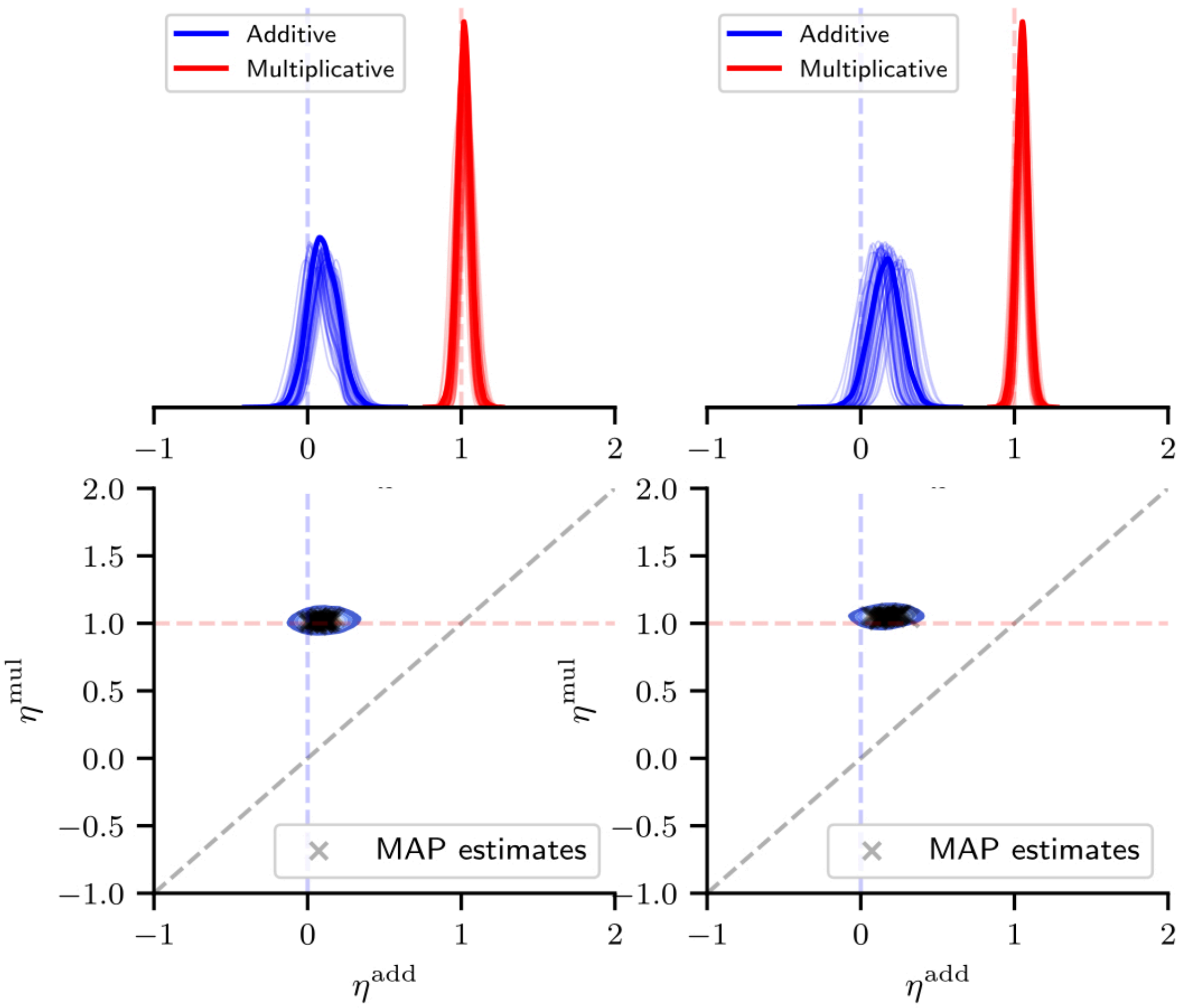

Figure S.13 | Bivariate distribution of posterior risk preference parameter for both additive and multiplicative conditions. Left, 1st half of decision trials, **right**, 2nd half of trials. Fiducials indicate theoretical predictions with blue indicating time optimal strategy for the additive condition, red the equivalent strategy for the multiplicative conditions, and the diagonal gray the predicted strategy under a dynamic invariant EUT model.

Reviewer #2: The results indicate a risk aversion parameter in the additive condition that is slightly positive, deviating from the theoretical optimum of zero.

Very marginally, the mean is within 1% at the group level.

Does further analysis of the choice data suggest whether this deviation is associated with specific properties of the gambles, such as the magnitude of potential losses, and could this be related to loss aversion?

The deviation is based on assessment of all of the gambles within the gamble pair space. We probe the relationship of risk preferences to differences between the conditions such as differences in variance or skew, and we find that no such effects are adequate explanations of the difference. We should point out that interpretation of the parameter space eta is not strictly linear, or have metric properties, due to the nature of the isoelastic equation, whereby different etas result in outputs that are not dimensionally consistent. As such it is hard to interpret whether the distance of the group mean to the additive optimum is meaningfully more of a deviation than that of the multiplicative optimum. That said, we have done extensive parameter recovery so we know that agents with this risk preference are reliably estimated. The estimation of risk preferences is done for all of the gambles, and thus it is not possible to isolate a subclass and attribute a risk preference to those. Risk preferences manifest over all the gambles under our modelling framework.

Regarding the cognitive mechanism, the experimental design included a real-time display of participants' wealth. What is your interpretation of the process underlying the observed adaptation, is it more likely an explicit process based on tracking wealth changes, or an implicit one based on learning the statistical properties of the gambles, and what effect would you predict if the wealth display were omitted?

Meder et al. ran a similar experiment in which wealth was not displayed and found quantitatively similar results. So whatever is driving the time optimal results, it cannot solely be dependent on wealth displays. We have now added a section to the discussion that reads:

The observed effect is unlikely to depend solely on the presence of an explicit wealth display: in our earlier study ~\cite{MederETAL2021}, wealth and gamble outcomes were not displayed, yet we observed quantitatively similar adaptation of the inferred transformation across dynamics (\fref{fig:replication}, lower row). This suggests that omitting the wealth display would not eliminate the core effect.

Finally, the study finds that while the wealth dynamic is a dominant factor, trait-like individual differences also persist. Could you elaborate on the potential nature of the interaction between these factors? For example, does the dynamic induce a consistent shift in each participant's baseline preference,

Yes—there is a highly consistent upward shift for all participants from additive to multiplicative, and the distribution of pairwise differences is uniformly positive (Fig. 8a–b)

or could the magnitude of the adaptation be moderated by individual characteristics? To help clarify these questions, a couple of additional analyses of the existing data or small experimental variations could be useful. A regression analysis could test whether the deviation from optimal behavior in the additive condition is disproportionately influenced by gambles with high-magnitude potential losses.

The reviewer suggests analyzing whether the magnitude of the context-dependent shift is larger for participants with greater "baseline" risk preference. While this is a natural idea, we do not view such analyses as diagnostically interpretable when the proposed predictor is one of the components of the change score. Specifically, correlating a difference score with either additive or multiplicative risk preferences induces a part–whole dependency (mathematical coupling), such that large correlations can arise mechanically from the shared term rather than reflecting a psychologically meaningful moderation of adaptation. In this setting, the more direct and interpretable question—whether trait-like individual differences persist across dynamics—is addressed by the cross-condition association between the two risk preference estimates themselves. Accordingly, we report the correlation between additive and multiplicative etas, which directly quantifies rank-order stability across environments and therefore captures the persistence of individual differences without the interpretational confound introduced by change-score coupling. On this basis, we do not pursue additional "change vs. baseline" regressions, as they would not add a clean inference beyond the correlation analysis already presented.

To explore the interaction between traits and dynamics, an analysis could examine whether the magnitude of the adaptive shift in risk aversion (the difference between the multiplicative and additive parameters for each individual) correlates with scores from the collected risk-propensity or personality, which could help identify traits associated with greater sensitivity to the environmental context.

Yes we have added the results of this analysis to Appendix D

Have the authors made all data and (if applicable) computational code underlying the findings in their manuscript fully available?
The PLOS Data policy requires authors to make all data and code underlying the findings described in their manuscript fully available without restriction, with rare exception (please refer to the Data Availability Statement in the manuscript PDF file). The data and code should be provided as part of the manuscript or its supporting information, or deposited to a public repository. For example, in addition to summary statistics, the data points behind means, medians and variance measures should be available. If there are restrictions on publicly sharing data or code —e.g. participant privacy or use of data from a third party—those must be specified.

Reviewer #1: Yes

Reviewer #2: Yes

PLOS authors have the option to publish the peer review history of their article (what does this mean?). If published, this will include your full peer review and any attached files.

If you choose "no", your identity will remain anonymous but your review may still be made public.

Do you want your identity to be public for this peer review? For information about this choice, including consent withdrawal, please see our Privacy Policy.

Reviewer #1: No

Reviewer #2: No

[NOTE: If reviewer comments were submitted as an attachment file, they will be attached to this email and accessible via the submission site. Please log into your account, locate the manuscript record, and check for the action link "View Attachments". If this link does not appear, there are no attachment files.]

Figure resubmission:

While revising your submission, we strongly recommend that you use PLOS's NAAS tool (https://ngplosjournals.pagemajik.ai/artanalysis) to test your figure files. NAAS can convert your figure files to the TIFF file type and meet basic requirements (such as print size, resolution), or provide you with a report on issues that do not meet our requirements and that NAAS cannot fix.

After uploading your figures to PLOS's NAAS tool - https://ngplosjournals.pagemajik.ai/artanalysis, NAAS will process the files provided and display the results in the "Uploaded Files" section of the page as the processing is complete. If the uploaded figures meet our requirements (or NAAS is able to fix the files to meet our requirements), the figure will be marked as "fixed" above. If NAAS is unable to fix the files, a red "failed" label will appear above. When NAAS has confirmed that the figure files meet our requirements, please download the file via the download option, and include these NAAS processed figure files when submitting your revised manuscript.

Reproducibility:

To enhance the reproducibility of your results, we recommend that authors of applicable studies deposit laboratory protocols in protocols.io, where a protocol can be assigned its own identifier (DOI) such that it can be cited independently in the future. Additionally, PLOS ONE offers an option to publish peer-reviewed clinical study protocols. Read more information on sharing protocols at
https://plos.org/protocols?utm_medium=editorial-email&utm_source=authorletters&utm_campaign=protocols
