# Supplementary material for: Ergodicity transformations predict human decision-making under risk
Source: PLoS Comput Biol. 2026 Jul 20;22(7):e1014409. doi: 10.1371/journal.pcbi.1014409 (PMC13472505; doi:10.1371/journal.pcbi.1014409)
Supplement: Supplementary file 3 [file pcbi.1014409.s003.docx]

We thank the reviewer for the two remaining points. Our responses are below. All corresponding edits are implemented in the tracked-changes manuscript; the Methods and Appendix D paragraphs referred to here are marked up there.

**Comment 1.**

*The authors stated that there were “~15 repetitions” for each image during the learning phase, yet in the methods as well as the results sections, they read “The learning task began with 45 learning trials”, and since the wealth was reset 3 times, that yielded ~15 repetitions for each image? During learning, did the images appear in an interleaved manner? or subjects have to learn ~15 trials for one image before moving to the next image? Were the multiplicative and additive learning phases separated in different blocks? Given the working load of 18 images and varying wealth amount, the “No-brainer” choices aren’t “non-brainer” after all (subjects had to remember their wealth levels since the current wealth was not displayed while they made no-brainer decisions, since the comparison across the multiplicative and additive images critically depended on the current wealth level).*

Images were interleaved within each learning run: the 45-trial sequence was a random uniform sampling with replacement of the nine images assigned to that session. Each session comprised three such runs, each followed by 15 no-brainer trials and a wealth reset, giving 135 learning trials per session. The additive and multiplicative conditions were separate sessions — not interleaved blocks — with independent nine-image sets (Ωₐ, Ωₘ) and a mandatory >10-minute break between them; the “18 images” is therefore the union across sessions, while the working-memory load at any one time is nine. The no-brainer task is solvable without tracking absolute wealth because (i) the ranking of images within a session is monotonic in wealth, so learning the image order is sufficient, and (ii) no-brainer responses do not update wealth. A direct learning-reliability check is already reported in Appendix D (no systematic difference in no-brainer accuracy between conditions; BF₀₁ = 7.3, Fig. S.12). Two clarifying sentences have been added to the Methods (Learning task; No-brainer task) in the tracked-changes manuscript.

**Comment 2.**

*During the actual decision phase, were the image pairs on the left and right randomly assigned? If yes, then the additional reinforcement heuristic analysis might not be convincing enough. The winning/losing of the previous trial might reinforce/diminish subjects’ risk attitude (instead of the left/right screen position adherence). More appropriate analyses might be either to the stay ratio of the winning image in the previous trial (if the image did re-appear in the current trial), or to the stay ratio of risk/variance-congruent image pair for the current trial (suppose subject chose the more risky/higher variance image pair in the last trial and won, then he/she was more likely to make the same risky/high variance choice in the current trial).*

The reviewer is correct: image pairs were randomly assigned to left/right positions, so the original side-based staying analysis was uninformative about reinforcement. We have rewritten the Reinforcement heuristic subsection of Appendix D to run the analysis at the level of the reviewer’s proposed features of the chosen gamble. Staying behaviour is now tested along three features of the previous trial: (i) side, (ii) gamble variance, and (iii) gamble variance conditional on having chosen the higher-variance gamble on the previous trial — this last test directly targets the reviewer’s specific example. For each feature and each condition we compute the per-participant paired difference between rewarded and non-rewarded previous trials on logit-transformed proportions, test it with a paired t-test, and report a default-prior Bayes factor (BF₀₁) against the null of no reward effect (six tests total; 3 features × 2 conditions). Paired differences are centred on zero and Bayes factors favour the null in all cases; the conditional variance test is likewise indistinguishable from zero, with Bayes-factor evidence for the null in the additive session and inconclusive evidence for a difference from 0 in the multiplicative session. Fig. S.11 has been replaced (below) to show these results, and the Reinforcement heuristic paragraph in Appendix D has been rewritten accordingly. We also removed the statistics of the win-stay-lose-shift analyses against 0.5 introduced in the last revision, as these were conducted using non-transformed probabilities and the test of differences between reward outcomes is more meaningful than testing against random outcomes.

**Figure S.11.** Strip-plots of paired differences in the logit-transformed stay probabilities between previously rewarded and non-rewarded trials, with paired t-test statistics (mean and 95% confidence intervals). Left panel: the three hypotheses tested per session. Left: does reward on the previous trial affect whether participants choose the same side on the current trial. Middle: does reward on the previous trial affect whether participants choose the same gamble variance on the current trial. Right: does reward on the previous trial affect whether participants continue choosing the higher-variance gamble, given they selected a high-variance gamble on the previous trial. Right panel: zoomed-out version of the high-variance plot, as some values fall outside the axis limits of the left panel.
