# Supplementary material for: Ergodicity transformations predict human decision-making under risk
Source: PLoS Comput Biol. 2026 Jul 20;22(7):e1014409. doi: 10.1371/journal.pcbi.1014409 (PMC13472505; doi:10.1371/journal.pcbi.1014409)
Supplement: Peer review history

# Peer review: Decision Letter 0

**DOI:** 10.1371/journal.pcbi.1014409.r001

**Authors:** Natalia L. Komarova, Jian Liu

19 Nov 2025

PCOMPBIOL-D-25-01780

Ergodicity transformations predict human decision-making under risk

PLOS Computational Biology

Dear Dr. Hulme,

Thank you for submitting your manuscript to PLOS Computational Biology. After careful consideration, we feel that it has merit but does not fully meet PLOS Computational Biology's publication criteria as it currently stands. Therefore, we invite you to submit a revised version of the manuscript that addresses the points raised during the review process.

Please submit your revised manuscript by Jan 19 2026 11:59PM. If you will need more time than this to complete your revisions, please reply to this message or contact the journal office at ploscompbiol@plos.org. When you're ready to submit your revision, log on to https://www.editorialmanager.com/pcompbiol/ and select the 'Submissions Needing Revision' folder to locate your manuscript file.

Please include the following items when submitting your revised manuscript:

* A rebuttal letter that responds to each point raised by the editor and reviewer(s). You should upload this letter as a separate file labeled 'Response to Reviewers'. This file does not need to include responses to formatting updates and technical items listed in the 'Journal Requirements' section below.

* A marked-up copy of your manuscript that highlights changes made to the original version. You should upload this as a separate file labeled 'Revised Manuscript with Track Changes'.

* An unmarked version of your revised paper without tracked changes. You should upload this as a separate file labeled 'Manuscript'.

If you would like to make changes to your financial disclosure, competing interests statement, or data availability statement, please make these updates within the submission form at the time of resubmission. Guidelines for resubmitting your figure files are available below the reviewer comments at the end of this letter

We look forward to receiving your revised manuscript.

Kind regards,

Jian Liu

Academic Editor

PLOS Computational Biology

Natalia Komarova

Section Editor

PLOS Computational Biology

**Additional Editor Comments:**

The manuscript requires significant revisions to strengthen its contribution. The relationship to previous work needs clarification to better highlight the novel contribution of this study. The rationale for the chosen design requires a more detailed justification. Additional analysis would help to fully support the conclusions drawn.

**Journal Requirements:**

1) We ask that a manuscript source file is provided at Revision. Please upload your manuscript file as a .doc, .docx, .rtf or .tex. If you are providing a .tex file, please upload it under the item type u2018LaTeX Source Fileu2019 and leave your .pdf version as the item type u2018Manuscriptu2019.

2) Please provide an Author Summary. This should appear in your manuscript between the Abstract (if applicable) and the Introduction, and should be 150-200 words long. The aim should be to make your findings accessible to a wide audience that includes both scientists and non-scientists. Sample summaries can be found on our website under Submission Guidelines:

https://journals.plos.org/ploscompbiol/s/submission-guidelines#loc-parts-of-a-submission

3) Please upload all main figures as separate Figure files in .tif or .eps format. For more information about how to convert and format your figure files please see our guidelines:

https://journals.plos.org/ploscompbiol/s/figures

4) We notice that your supplementary Figures, Tables, and information are included in the manuscript file. Please remove them and upload them with the file type 'Supporting Information'. Please ensure that each Supporting Information file has a legend listed in the manuscript after the references list.

5) Please amend your detailed Financial Disclosure statement. This is published with the article. It must therefore be completed in full sentences and contain the exact wording you wish to be published.

- State the initials, alongside each funding source, of each author to receive each grant. For example: "This work was supported by the National Institutes of Health (####### to AM; ###### to CJ) and the National Science Foundation (###### to AM)."

- State what role the funders took in the study. If the funders had no role in your study, please state: "The funders had no role in study design, data collection and analysis, decision to publish, or preparation of the manuscript.".

If you did not receive any funding for this study, please simply state: u201cThe authors received no specific funding for this work.u201d

**Reviewers' comments:**

Reviewer's Responses to Questions

**Comments to the Authors:**

**Please note here if the review is uploaded as an attachment.**

Reviewer #1: The paper by Skjold et al. aimed to provide a mechanistic explanation for the context specific risk preferences by participants. Namely, during the additive and multiplicative environments, subjects don't have stable risk preference, instead, their risk attitudes are driven by accruing wealth in the fastest manner through ergodicity transformation. They provided model comparison results between the standard utility (EUT) model and the ergodicity transformation (EE) model.

My concerns regarding this manuscripts are listed below:

1. As the author correctly pointed out, the comparison between the additive and multiplicative decision environments have been tested before and similar results were obtained (Meder et al., 2021), so I'm not fully convinced whether the current manuscript provided enough novelty to the extant literature.

2. In the discussion, the authors pointed out that one of the drawbacks of the Meder et al. (2021) paper was that "wealth and gamble outcomes were hidden from the participants" and to avoid that, the current manuscript provided participants with the accumulated wealth for each trial. However, by providing feedbacks, the decision trials are not solely driven by participants risk attitudes (either context dependent or independent) anymore. Feedbacks after each trials would act as strong incentive to influence their subsequent trials. One way to test this is to look at subjects' choice preference after winning or losing in the previous trial. To me, not providing feedback in the original paper was a well thought-out move rather than a drawback.

3. Have the author tested how reliable/accurate about the outcomes from the learning phases? Given there were only 5 repetitions for each image and subjects' wealth level in each trial is likely to be different, participants might turn out to have a more vague estimation of the multiplicative factor than that of the the additive amount for the images. Previous research in cognitive neuroscience have shown how the belief of the environment uncertainty might have an impact on the speed of learning (e.g. McGuire et al., 2014 Neuron) and this might also affect the difference in the later comparison between the additive and multiplicative comparisons.

4. The authors did perform exploratory analysis to control for the effects of potential confounders such as variance in wealth, or log wealth. However, since the range of wealth (or monetary prospects) in the decision tasks are dramatically different between the additive and multiplicative contexts and it's well established that the utility is marginally decreasing, I'm concerned whether the results and estimated parameters are reliable enough if candidate utility models are also included and compared against. For example, what about constructing a EUT model that takes into account the law of diminishing marginal return (LDMR) or a model that considers the contribution of learning components in the pre-assumed "decision" task?

Reviewer #2: The results indicate a risk aversion parameter in the additive condition that is slightly positive, deviating from the theoretical optimum of zero. Does further analysis of the choice data suggest whether this deviation is associated with specific properties of the gambles, such as the magnitude of potential losses, and could this be related to loss aversion?

Regarding the cognitive mechanism, the experimental design included a real-time display of participants' wealth. What is your interpretation of the process underlying the observed adaptation, is it more likely an explicit process based on tracking wealth changes, or an implicit one based on learning the statistical properties of the gambles, and what effect would you predict if the wealth display were omitted?

Finally, the study finds that while the wealth dynamic is a dominant factor, trait-like individual differences also persist. Could you elaborate on the potential nature of the interaction between these factors? For example, does the dynamic induce a consistent shift in each participant's baseline preference, or could the magnitude of the adaptation be moderated by individual characteristics?

To help clarify these questions, a couple of additional analyses of the existing data or small experimental variations could be useful. A regression analysis could test whether the deviation from optimal behavior in the additive condition is disproportionately influenced by gambles with high-magnitude potential losses. To explore the interaction between traits and dynamics, an analysis could examine whether the magnitude of the adaptive shift in risk aversion (the difference between the multiplicative and additive parameters for each individual) correlates with scores from the collected risk-propensity or personality, which could help identify traits associated with greater sensitivity to the environmental context.

**********

**Have the authors made all data and (if applicable) computational code underlying the findings in their manuscript fully available?**

The PLOS Data policy requires authors to make all data and code underlying the findings described in their manuscript fully available without restriction, with rare exception (please refer to the Data Availability Statement in the manuscript PDF file). The data and code should be provided as part of the manuscript or its supporting information, or deposited to a public repository. For example, in addition to summary statistics, the data points behind means, medians and variance measures should be available. If there are restrictions on publicly sharing data or code —e.g. participant privacy or use of data from a third party—those must be specified.

Reviewer #1: Yes

Reviewer #2: Yes

**********

PLOS authors have the option to publish the peer review history of their article (what does this mean?). If published, this will include your full peer review and any attached files.

If you choose “no”, your identity will remain anonymous but your review may still be made public.

**Do you want your identity to be public for this peer review?**  For information about this choice, including consent withdrawal, please see our Privacy Policy.

Reviewer #1: No

Reviewer #2: No

[NOTE: If reviewer comments were submitted as an attachment file, they will be attached to this email and accessible via the submission site. Please log into your account, locate the manuscript record, and check for the action link "View Attachments". If this link does not appear, there are no attachment files.]

**Figure resubmission:**

While revising your submission, we strongly recommend that you use PLOS’s NAAS tool (https://ngplosjournals.pagemajik.ai/artanalysis) to test your figure files. NAAS can convert your figure files to the TIFF file type and meet basic requirements (such as print size, resolution), or provide you with a report on issues that do not meet our requirements and that NAAS cannot fix.

After uploading your figures to PLOS’s NAAS tool - https://ngplosjournals.pagemajik.ai/artanalysis, NAAS will process the files provided and display the results in the "Uploaded Files" section of the page as the processing is complete. If the uploaded figures meet our requirements (or NAAS is able to fix the files to meet our requirements), the figure will be marked as "fixed" above. If NAAS is unable to fix the files, a red "failed" label will appear above. When NAAS has confirmed that the figure files meet our requirements, please download the file via the download option, and include these NAAS processed figure files when submitting your revised manuscript.

**Reproducibility:**

To enhance the reproducibility of your results, we recommend that authors of applicable studies deposit laboratory protocols in protocols.io, where a protocol can be assigned its own identifier (DOI) such that it can be cited independently in the future. Additionally, PLOS ONE offers an option to publish peer-reviewed clinical study protocols. Read more information on sharing protocols at https://plos.org/protocols?utm_medium=editorial-email&utm_source=authorletters&utm_campaign=protocols

# Author comment: Author response to Decision Letter 1

**DOI:** 10.1371/journal.pcbi.1014409.r002

3 Feb 2026

Attachment Submitted filename: ergEx_ResponseToReviewers.pdf

# Peer review: Decision Letter 1

**DOI:** 10.1371/journal.pcbi.1014409.r003

**Authors:** Natalia L. Komarova, Jian Liu

24 Feb 2026

PCOMPBIOL-D-25-01780R1

Ergodicity transformations predict human decision-making under risk

PLOS Computational Biology

Dear Dr. Hulme,

Thank you for submitting your manuscript to PLOS Computational Biology. After careful consideration, we feel that it has merit but does not fully meet PLOS Computational Biology's publication criteria as it currently stands. Therefore, we invite you to submit a revised version of the manuscript that addresses the points raised during the review process.

Please submit your revised manuscript by Apr 26 2026 11:59PM. If you will need more time than this to complete your revisions, please reply to this message or contact the journal office at ploscompbiol@plos.org. When you're ready to submit your revision, log on to https://www.editorialmanager.com/pcompbiol/ and select the 'Submissions Needing Revision' folder to locate your manuscript file.

Please include the following items when submitting your revised manuscript:

* A letter that responds to each point raised by the editor and reviewer(s). You should upload this letter as a separate file labeled 'Response to Reviewers'. This file does not need to include responses to formatting updates and technical items listed in the 'Journal Requirements' section below.

* A marked-up copy of your manuscript that highlights changes made to the original version. You should upload this as a separate file labeled 'Revised Manuscript with Track Changes'.

* An unmarked version of your revised paper without tracked changes. You should upload this as a separate file labeled 'Manuscript'.

If you would like to make changes to your financial disclosure, competing interests statement, or data availability statement, please make these updates within the submission form at the time of resubmission. Guidelines for resubmitting your figure files are available below the reviewer comments at the end of this letter.

We look forward to receiving your revised manuscript.

Kind regards,

Jian Liu

Academic Editor

PLOS Computational Biology

Natalia Komarova

Section Editor

PLOS Computational Biology

**Additional Editor Comments:**

There are minor questions regarding the clarification of the methods and analysis. Please address these points appropriately in the revised manuscript.

**Journal Requirements:**

1) We notice that your supplementary Figures, Tables, and information are included in the manuscript file. Please remove them and upload them with the file type 'Supporting Information'. Please ensure that each Supporting Information file has a legend listed in the manuscript after the references list.

2) Please amend your detailed Financial Disclosure statement. This is published with the article. It must therefore be completed in full sentences and contain the exact wording you wish to be published.

- State the initials, alongside each funding source, of each author to receive each grant. For example: "This work was supported by the National Institutes of Health (####### to AM; ###### to CJ) and the National Science Foundation (###### to AM)."

- State what role the funders took in the study. If the funders had no role in your study, please state: "The funders had no role in study design, data collection and analysis, decision to publish, or preparation of the manuscript.".

If you did not receive any funding for this study, please simply state: u201cThe authors received no specific funding for this work.u201d

**Reviewers' comments:**

Reviewer's Responses to Questions

**Comments to the Authors:**

**Please note here if the review is uploaded as an attachment.**

Reviewer #1: I thank the authors for their thorough feedbacks and additional analyses performed on the revised manuscript. I have only two remaining questions:

1. The authors stated that there were "~15 repetitions" for each image during the learning phase, yet in the methods as well as the results sections, they read "The learning task began with 45 learning trials", and since the wealth was reset 3 times, that yielded ~15 repetitions for each image? During learning, did the images appear in an interleaved manner? or subjects have to learn ~15 trials for one image before moving to the next image? Were the multiplicative and additive learning phases separated in different blocks? Given the working load of 18 images and varying wealth amount, the "No-brainer" choices aren't "non-brainer" after all (subjects had to remember their wealth levels since the current wealth was not displayed while whey made no-brainer decisions, since the comparison across the multiplicative and additive images critically depended on the current wealth level).

2. During the actual decision phase, were the image pairs on the left and right randomly assigned? If yes, then the additional reinforcement heuristic analysis might not be convincing enough. The winning/losing of the previous trial might reinforce/diminish subjects' risk attitude (instead of the left/right screen position adherence). More appropriate analyses might be either to the stay ratio of the winning image in the previous trial (if the image did re-appear in the current trial), or to the stay ratio of risk/variance-congruent image pair for the current trial (suppose subject chose the more risky/higher variance image pair in the last trial and won, then he/she was more likely to make the same risky/high variance choice in the current trial).

**********

**Have the authors made all data and (if applicable) computational code underlying the findings in their manuscript fully available?**

The PLOS Data policy requires authors to make all data and code underlying the findings described in their manuscript fully available without restriction, with rare exception (please refer to the Data Availability Statement in the manuscript PDF file). The data and code should be provided as part of the manuscript or its supporting information, or deposited to a public repository. For example, in addition to summary statistics, the data points behind means, medians and variance measures should be available. If there are restrictions on publicly sharing data or code —e.g. participant privacy or use of data from a third party—those must be specified.

Reviewer #1: Yes

**********

PLOS authors have the option to publish the peer review history of their article (what does this mean?). If published, this will include your full peer review and any attached files.

If you choose “no”, your identity will remain anonymous but your review may still be made public.

**Do you want your identity to be public for this peer review?**  For information about this choice, including consent withdrawal, please see our Privacy Policy.

Reviewer #1: No

[NOTE: If reviewer comments were submitted as an attachment file, they will be attached to this email and accessible via the submission site. Please log into your account, locate the manuscript record, and check for the action link "View Attachments". If this link does not appear, there are no attachment files.]

**Figure resubmission:**

-->While revising your submission, we strongly recommend that you use PLOS’s NAAS tool (https://ngplosjournals.pagemajik.ai/artanalysis) to test your figure files. NAAS can convert your figure files to the TIFF file type and meet basic requirements (such as print size, resolution), or provide you with a report on issues that do not meet our requirements and that NAAS cannot fix.

After uploading your figures to PLOS’s NAAS tool - https://ngplosjournals.pagemajik.ai/artanalysis, NAAS will process the files provided and display the results in the "Uploaded Files" section of the page as the processing is complete. If the uploaded figures meet our requirements (or NAAS is able to fix the files to meet our requirements), the figure will be marked as "fixed" above. If NAAS is unable to fix the files, a red "failed" label will appear above. When NAAS has confirmed that the figure files meet our requirements, please download the file via the download option, and include these NAAS processed figure files when submitting your revised manuscript.

**Reproducibility:**

To enhance the reproducibility of your results, we recommend that authors of applicable studies deposit laboratory protocols in protocols.io, where a protocol can be assigned its own identifier (DOI) such that it can be cited independently in the future. Additionally, PLOS ONE offers an option to publish peer-reviewed clinical study protocols. Read more information on sharing protocols at https://plos.org/protocols?utm_medium=editorial-email&utm_source=authorletters&utm_campaign=protocols

# Author comment: Author response to Decision Letter 2

**DOI:** 10.1371/journal.pcbi.1014409.r004

12 May 2026

Attachment Submitted filename: reply-to-reviewers.docx

# Peer review: Decision Letter 2

**DOI:** 10.1371/journal.pcbi.1014409.r005

**Authors:** Natalia L. Komarova, Jian Liu

4 Jun 2026

Dear Hulme,

We are pleased to inform you that your manuscript 'Ergodicity transformations predict human decision-making under risk' has been provisionally accepted for publication in PLOS Computational Biology.

Before your manuscript can be formally accepted you will need to complete some formatting changes, which you will receive in a follow up email. A member of our team will be in touch with a set of requests.

Please note that your manuscript will not be scheduled for publication until you have made the required changes, so a swift response is appreciated.

IMPORTANT: The editorial review process is now complete. PLOS will only permit corrections to spelling, formatting or significant scientific errors from this point onwards. Requests for major changes, or any which affect the scientific understanding of your work, will cause delays to the publication date of your manuscript.

Should you, your institution's press office or the journal office choose to press release your paper, you will automatically be opted out of early publication. We ask that you notify us now if you or your institution is planning to press release the article. All press must be co-ordinated with PLOS.

Thank you again for supporting Open Access publishing; we are looking forward to publishing your work in PLOS Computational Biology.

Best regards,

Jian Liu

Academic Editor

PLOS Computational Biology

Natalia Komarova

Section Editor

PLOS Computational Biology

***********************************************************

Reviewer's Responses to Questions

**Comments to the Authors:**

**Please note here if the review is uploaded as an attachment.**

Reviewer #1: I don't have further questions and would like to thank the authors for introducing the underappreciated ergodicity concept into deicison making under risk in behavioral and psychology studies.

**********

**Have the authors made all data and (if applicable) computational code underlying the findings in their manuscript fully available?**

The PLOS Data policy requires authors to make all data and code underlying the findings described in their manuscript fully available without restriction, with rare exception (please refer to the Data Availability Statement in the manuscript PDF file). The data and code should be provided as part of the manuscript or its supporting information, or deposited to a public repository. For example, in addition to summary statistics, the data points behind means, medians and variance measures should be available. If there are restrictions on publicly sharing data or code —e.g. participant privacy or use of data from a third party—those must be specified.

Reviewer #1: Yes

**********

PLOS authors have the option to publish the peer review history of their article (what does this mean?). If published, this will include your full peer review and any attached files.

If you choose “no”, your identity will remain anonymous but your review may still be made public.

**Do you want your identity to be public for this peer review?**  For information about this choice, including consent withdrawal, please see our Privacy Policy.

Reviewer #1: No

# Editor report: Acceptance letter

**DOI:** 10.1371/journal.pcbi.1014409.r006

**Authors:** Natalia L. Komarova, Jian Liu

PCOMPBIOL-D-25-01780R2

Ergodicity transformations predict human decision-making under risk

Dear Dr Hulme,

I am pleased to inform you that your manuscript has been formally accepted for publication in PLOS Computational Biology. Your manuscript is now with our production department and you will be notified of the publication date in due course.

The corresponding author will soon be receiving a typeset proof for review, to ensure errors have not been introduced during production. Please review the PDF proof of your manuscript carefully, as this is the last chance to correct any errors. Please note that major changes, or those which affect the scientific understanding of the work, will likely cause delays to the publication date of your manuscript.

Soon after your final files are uploaded, unless you have opted out, the early version of your manuscript will be published online. The date of the early version will be your article's publication date. The final article will be published to the same URL, and all versions of the paper will be accessible to readers.

For Research, Software, and Methods articles, you will receive an invoice from PLOS for your publication fee after your manuscript has reached the completed accept phase. If you receive an email requesting payment before acceptance or for any other service, this may be a phishing scheme. Learn how to identify phishing emails and protect your accounts at https://explore.plos.org/phishing.

Thank you again for supporting PLOS Computational Biology and open-access publishing. We are looking forward to publishing your work!

With kind regards,

Janani Seenivasan

PLOS Computational Biology | Carlyle House, Carlyle Road, Cambridge CB4 3DN | United Kingdom ploscompbiol@plos.org | Phone +44 (0) 1223-442824 | ploscompbiol.org | @PLOSCompBiol
